# Multicomponent Aging Al-Li-Based Alloys of the Latest Generation: Structural and Phase Transformations, Treatments, Properties, and Future Prospects

**DOI:** 10.3390/ma15124190

**Published:** 2022-06-13

**Authors:** Dmitriy Y. Rasposienko, Larisa I. Kaigorodova, Vladimir G. Pushin, Yurii M. Ustugov

**Affiliations:** 1 Mikheev Institute of Metal Physics, Ural Branch, Russian Academy of Sciences, 620108 Ekaterinburg, Russia; likaigorodova@mail.ru (L.I.K.); ustyugov@imp.uran.ru (Y.M.U.); 2 Department of Metallurgy and Metal Science, Yeltsin Ural Federal University, 620002 Ekaterinburg, Russia

**Keywords:** aluminum lithium alloys (Al-Li), severe (mega) plastic deformation (SPD), high pressure torsion (HPT), heat treatment (HT), aging, ultrafine-grained (UFG), nanocrystalline (NC) structure, phase transformation, mechanical properties

## Abstract

An overview of modern material science problems is presented for ultralightweight high-modulus commercial Al-Li-based alloys in historical retrospect. Numerous particular examples of the Soviet and Russian aviation whose various designs were made of these alloys confirm their successful innovative potential. The key regularities of multicomponent alloying are discussed for the master alloys and modern commercial Al-Li-based alloys of the latest generation; the features typical of their microstructures, phase composition, and properties formed during aging are analyzed. The main mechanisms of phase formation are generalized for standard thermal and thermomechanical treatments. Recent original achievements have been obtained in designing of unique structural and phase transformations in these commercial alloys by means of methods of severe plastic deformations followed by heat treatment and storage. Using the example of three Russian commercial alloys of last generation, the basic principles of creating and controlling an ultrafine-grained structure, the origin and growth of stable nanophases of various types and chemical composition that determine the physicomechanical properties of alloys are established.

## 1. Introduction

Ultralow-weight Al-Li-based alloys are of wide scientific and commercial interest due to the excellent combination of physical and mechanical properties: reduced density, increased modulus of elasticity, and specific strength in a wide range of temperatures from cryogenic to high. Currently, aluminum itself and its alloys are the second most common species among practically used metal materials after steels and iron-based alloys. Aluminum is widely used in electrical engineering, and its alloys, first of all, find wide application in the aerospace and rocket industry as structural materials for various purposes [1,2,3,4,5]. The history of the design and development of these alloys began only in the XX century with the discovery in 1906 by Alfred Wilm of the aging effect in the alloy Duralumin Al–4%Cu–0.5%Mg–0.5%Mn (in wt%) [3]. In 1924, Germany also developed for the first time a Li-containing alloy Scleron Al–12%Zn–2%Cu–0.5%Mn–0.1%Li with better properties than duralumin [3]. Despite the low Li content (0.1%), the technological difficulties of manufacturing and, as a result, the high self-cost excluded the development of Al-Li-based alloys for practical purposes for 30 years. It was only in the 1950s of the twentieth century that research was started again and at the same time alloys of the Al-Li-Cu system of grades VAD23 in the Soviet Union and 2020 in the USA, similar in content to Li and Cu, were designed, which turned out to be promising for industrial use.

However, as experimental studies have shown, the alloy VAD23, like all Li-alloyed aluminum alloys, was rapidly oxidized in the melt and showed an increased tendency to hydrogen saturation and to the formation of casting cracks, which required the development of special methods for their smelting and casting [2,5]. The work carried out in the Soviet Union at existing equipment made it possible to manage the melting and casting of the VAD23 alloy in a continuous way and to use it in rocket engineering and in the design of the Tu-144 supersonic passenger aircraft [5]. Soon, the created alloy 1420 based on the Al-Li-Mg system was used in the manufacture of a number of Yak-36, Yak-38, and Mig-29 aircraft [5,6]. On the contrary, the company Alcoa (USA) has stopped research and production of semifinished products of Li-containing alloy 2020 due to the complexity of the ingot production technology and their brittleness [2].

Already at the initial stage of the development of aluminum alloys in the USSR, the key factor determining the success of their production, systematic research, and testing of properties, as well as industrial production and widespread application, was the creation of a specialized state scientific and technological complex. The scientific–creative association between (i) the Federal State Unitary Enterprise “All-Russian Research Institute of Aviation Materials (VIAM)” and (ii) the aircraft design, metallurgical, and aircraft manufacturing enterprises is still in operation. Aluminum alloys developed at the Institute VIAM under the scientific supervision of I. N. Friedlander were used in the designs of all Soviet and Russian aircraft, including passenger and transport An-22, An-124 Ruslan, An-148, An-225 Mriya, Il-86, Il-96, Tu-144, Tu-154,Tu-204, MS-21, Superjet, Yak-42, amphibious aircraft Be-103 and Be-200, strategic bombers Tu-16, Tu-95, Tu-160, fighters MiG-15, MiG-23, MiG-29, Su-30, Su-34, Su-35, Su-37 Yak-141, and others, as well as solid- and liquid-fuel short- and medium-range and intercontinental missiles [5,6,7,8,9,10,11,12,13,14,15,16].

Of course, a separate important global trend of modern development was the research on the development of ultralow-weight Al-Li-based alloys, which has included the study of both model binary and alloyed master-alloys and commercially employed alloys [2,3,4,5,6,7,8,9,10,11,12,13,14,15,16].

However, their applications are still limited because of shortcomings of thermal stability, brittleness, and mechanical strength, which are closely related to the chemical composition and microstructural and phase characteristics of the alloys. Aging Al-Li-based alloys are distinguished by their complicated structure–phase transformations when being heat- (HT) and thermomechanically treated (TMT), which exert an influence on the subgrain and grain sizes, congregation of secondary phases or impurities along the grain boundaries, and precipitations within grains. Attempts have been made to overcome these drawbacks by the means of proper multicomponent additions, alternative processing routes, and optimizing of the HT and TMT conditions. The present article shall deal with the current status of research and commercialization of Al-Li-based aging alloys and dwell upon the future directions in which investigations should be targeted into various commercial uses.

## 2. Materials and Methods

The studies were carried out on high-strength aging multicomponent commercial alloys of the Al-Li-Cu system of the second and third generations of Russian production, namely, the alloys 1441, 1450, 1461, and 1469 (Table 1 and Table 2).

The alloy 1450 of the Al-Cu-Li-Zr system belongs to high-strength aluminum-lithium alloys of a reduced density (2600 kg/m^3^), and it was developed to replace the V95-type alloy of the Al-Zn-Cu-Mg system [5,6]. The alloy 1450 is used for the production of wrought semifinished products intended for the manufacture of parts of wing top coverings (plates, sheets), stringers (bent ones—sheet and pressed), beams, struts, and other elements of the fuselage and wing of modern aircraft and other highly loaded structures working mainly on compression. Pressed semifinished products made of alloy 1450 have found use instead of the alloy V95. (The Russian nomenclature is transliterated).

Table 1 shows that the alloy 1450 corresponds to the second-generation alloys containing 2 or more wt.% Li. The main disadvantage of the alloys of this class is their reduced ductility. In the 1450 alloys with a microcrystalline structure, it is due to the localization of stresses near the grain boundaries, resulting from the interaction of moving dislocations with the precipitations of the strengthening metastable phase δ’ (Al_3_Li), ordered by the type *L*1_2_ [17], and with the precipitations of the stable phase T_1_ (Al_2_CuLi), localized along the grain boundaries in the form of a continuous film [18,19,20]. The number density of such precipitates can be reduced both by activating their nucleation and growth inside the grains, and by size refinement of the grain structure. Another effective way of improving the structure is additional alloying of the alloy, which can affect both the grain structure and the decomposition of the supersaturated solid solution. Our studies of the effect of severe plastic deformation (SPD) on the structural and phase transformations were performed both on the standard alloy 1450 and, mainly, on the 1450 alloy modified additionally with Sc and Mg additives (Table 1 and Table 2) [5,6,7].

The choosing of the alloying elements already in the second-generation alloys was due to the fact that Zr and Sc are effective modifiers of the cast structure, and their addition contributes to an increase in the recrystallization temperature of the deformed semifinished products of these aluminum alloys, and Mg changes the solubility of the main alloying components (i.e., lithium and copper) in aluminum [21,22].

The alloy 1441 is a corrosion-resistant alloy; it shows excellent handling (processibility) properties during cold and hot deformation, being the most technologically advanced and less alloyed in Cu and Li among all modern Al-Li-based alloys. It is characterized by low anisotropy of properties and is of high resource with increased fatigue and crack resistance characteristics [8,10]. Clad sheets up to 0.3 mm thick are used in the cold-rolled state and have found application, including the use in the design of seaplanes.

The alloy 1461 is a high-strength, weldable, corrosion-resistant alloy developed in the capacity of an alternative to the V95 alloy [13]. Compared with the latter, it has a reduced density (2630 kg/m^3^) and an increased modulus of elasticity (79 GPa), increased strength, and heat resistance characteristics [13]. The development of the 1461 alloy was aimed at reducing the weight of the riveted or the welded structure by an amount of 8% and 15%, respectively. Various types of semifinished products (plates, pressed strips, profiles, sheets) are made from the alloy 1461. The process of manufacturing semifinished products, their HT and TMT, as well as the technological deformation of the straightening (leveling) and temper rolling strongly affect the structural, phase state, and properties of the multiphase alloy.

The promising high-strength alloy 1469 of the Al-Cu-Li-Mg system, developed at VIAM, is the first silver-alloyed Al-Li alloy in Russia [13,23]. This alloy belongs to the group of Al-Li-based alloys with a relatively high copper content (3.0–4.5 wt.%), lower Li content compared to the alloy 1450 (1.0 wt.%), and contains the additives Zr, Sc, Mg, and Ag (Table 1). The alloy has a lower density, an increased modulus of elasticity compared to the alloy V95, a successful combination of strength and plastic properties and is successfully used in industry for the manufacture of pressed and rolled semifinished products. Alloy 1469 is well welded to be promising for use in both aviation and aerospace engineering [24,25]. This alloy has a number of features in comparison with other Al-Li alloys, since one of its alloying components is silver, which is known to have a significant effect on the structural and phase transformations in aluminum alloys, and, accordingly, on their properties.

In our work, alloys with a conventional microcrystalline (MC) structure in the state of delivery and after heat treatment for maximum strength, as well as with the fine-grained (FG), the ultrafine-grained (UFG), and also with the nanocrystalline (NC) structure are studied. In the study of the alloys in the initial state, samples cut from sheets with a thickness of 2 mm were used. Before deformation, the alloys, due to the alloying of Zr and Sc, were characterized by an FG structure, mainly consisting of grains of 5–10 microns, although grains up to 50 microns in size were found in some parts of the samples. All grains, especially the larger ones (with a diameter of 10–50 microns), contained subgrains with small-angle boundaries at misorientation angles of no more than 2°, the sizes of the subgrains, as a rule, did not exceed 1–1.5 microns.

To obtain maximum strength and hardness, samples of all the alloys were tempered from 530 °C, 15 min in water and subjected to artificial aging, namely, the alloys: 1450—at 190 °C, 10 h; 1461 and 1469—at 160 °C, 32 h.

In order to obtain the UFG structure, hardened alloy samples were subjected to high-pressure torsion (HPT) in Bridgman anvils at room temperature (RT). Large-size samples for deformation were cut out from sheets 2-mm thick, in the form of disks with a diameter of 2 *r* = 15 mm and thickness *h*_0_ = 2 mm. The employed HPT regimes for the alloys 1441, 1450, 1469, and 1461 are listed in Table 3 and Table 4, respectively; for the alloy 1441, we used only HPT regime—*P* = 4 GPa, *n* = 5.

The formation of a recrystallized UFG structure after HPT was achieved by means of low-temperature isothermal anneals of the alloys at 190 and 150 °C, for 10 and 15 h, respectively.

When calculating the value of true logarithmic deformation ***e***, we employed the formula that takes into account the shear deformation and a decrease in the thickness of a sample in result of pressure application [26]:

(1) $$e=ln\left(\frac{2\pi Nr}{h}\right)+ln\left(\frac{h_{0}}{h}\right),$$

where *h*_0_ and *h* are the thickness of the sample before and after deformation, respectively.

The formation of the structure during HPT processing occurs under the influence of not only external but also internal stresses. At the same time, there is no rigid connection between the magnitude of the latter and the true deformations. This is confirmed by the possibility of forming a structure homogeneous over the diameter of the samples subjected to HPT. In this regard, when studying the evolution of microstructure during HPT, it is often more correct to consider the number of revolutions *n*, rather than the amount of deformation *e* calculated using analytical expressions.

Microstructural features of the materials were studied using electron transmission microscopes (TEM, Hillsboro, OR, USA) Tecnai G^2^ 30 Twin and Phillips CM30 Super Twin (Eindhoven, The Netherland) at accelerating voltages of 300 kV and JEM-200CX—at 160 kV. The study of the structure and the phase analysis were carried out by the TEM method using standard techniques of bright-field, dark-field images, and electron microdiffraction [27]. Linear dimensions of the structural elements were determined by direct measurements on the observation plane. For each alloy, plots of the size distributions of the nanoparticles and nanofragments were constructed, their average size and mean square-root deviations were calculated. At least 100 measurements of dimensions of structural elements were carried out during the construction of plots. Identification of the phases precipitated during the decomposition of a supersaturated solid solution was carried out by calculating their interplanar distances by the positions of additional reflections in electron diffraction patterns. The calculated interplanar distances were compared with the numerical data given in the international radiometric tables (JCPDS-ICDD). To establish the morphology and size distribution of each of the precipitated phases, dark-field images were analyzed in additional reflections from these phases in which they taken.

Specific features of the structure and decay of a supersaturated solid solution of the alloys were also studied using scanning electron microscopy (SEM) with attracting analytical methods of the energy dispersive microanalysis and backscattered electron diffraction (EBSD) in an SEM Quanta 200 Pegasus instrument (at an accelerating voltage of 30 kV). The use of the EBSD method made it possible to determine the crystallographic orientations of individual crystallites in the samples, identify phases, measure the angles of inclination of intergrain and subgrain boundaries, build maps of the grain orientations, and phase distributions over the sample surface.

Samples for the TEM, SEM, and XRD studies were prepared by abrasion on coarse-grained emery paper followed by finish mechanical polishing to obtain a visually smooth surface. Samples for TEM studies were further thinned by double-sided polishing on fine-grained emery paper down to 40–60 microns. To reduce surface mechanical stresses and obtain foils suitable for TEM investigations, electropolishing was performed in an electrolyte of the following composition: 23% perchloric acid (density—1600 kg/m^3^) and 77% ice acetic acid.

Mechanical properties (microhardness, modulus of elasticity, plasticity, and toughness) were measured by instrumental indentation on nanohardness testers Nanotest Micro Materials Ltd. Wrexham UK and FISHERSCOPE 2000 with a diamond Berkovich indenter and Vickers indenter, respectively. The tests were performed at a one-half (½) of the sample radius, at a loading of 150 and 500 mN.

The test samples were prepared by abrasion on emery paper followed by finish mechanical polishing to obtain flat parallel surfaces without surface irregularities. For each load, 10 indentations were made on the sample surface, at a distance of 50 microns from each other. To determine the hardness and the elastic modulus *E*, the Oliver–Pharr method was used [28]. The hardness of the sample, in units of Mayer (H_M_) and Vickers (HV), and the value of instrumental (HIT) hardness were calculated from the indentation depth of the indenter in accordance with ISO 14577; the reduced modulus of elasticity was calculated from the curve of unloading.

When determining the plasticity characteristics, a method was used to determine the work of deformation; using the loading/unloading curves, the plasticity characteristics were calculated as the ratio of the plastic deformation work to the full work when the indenter was introduced [29]:

(2) $$\delta_{A}=\frac{A_{p}}{A_{t}}=\frac{A_{p}}{A_{p}+A_{e}}=1-\frac{A_{e}}{A_{t}},$$

where *A_p_*, *A_e_*, and *A_t_* in each case is the work spent on the plastic, the elastic, and the total deformation during the intrusion of the indenter, respectively.

## 3. Results and Discussion

### 3.1. Classification of Al-Li-Based Alloys

The generations of commercial Al-Li-based alloys are presented in Table 5 in the world historical retrospective from the first alloys 2020 and 2023 to the latest modern alloys. A series of alloys developed in the 1970s–1980s is commonly referred to as Al-Li-based alloys of the second generation. As a rule, they are characterized by a feature that consist in alloying with two or more percent of Li, which made it possible to reduce the density of alloys by 7–10%, increase the modulus of elasticity by 10–15%, and ensure high fatigue durability (low growth rate of fatigue cracks). Unfortunately, the alloys also had a number of negative properties: low fracture toughness in the transverse direction of the products, low plasticity, and high anisotropy of strength properties [16]. Analysis of the main disadvantages of the second generation alloys and the reasons of their occurrence led to the further development of alloys with a lower Li content from 0.75% to 1.8%, which belong to the third generation. Most of these alloys are developed on the basis of the Al-Li-Cu system. In Russia, in VIAM, there also developed and introduced into practice the high-strength alloys 1461, 1469 [30,31,32,33,34], which are an alternative to the main structural alloys—of the Al-Cu-Mg and Al-Zn-Mg-Cu systems—of the types 2X24, 7075, and V95 [13,31].

Chronological analysis of the development of commercial alloys reveals the fundamental principles of their production. Firstly, three basic doping systems were selected: Al-Li-Cu, Al-Li-Mg, and Al-Li-Cu-Mg (Table 5). Secondly, progress in the combined alloying of Al-Li-based alloys with small additives of the following elements played a key role: first, in single—by Mn or Zr; then, in multicomponent—Zr + Sc, Zr + Ag, Zr + Mn + Zn, Zr + Sc + Zn, and Zr + Mn +Ag + Zn. In the capacity of a third important circumstance, it should be noted the constant search for solutions to optimize all alloying major and minor chemical components. At the same time, it can be seen that a new basic trend is being implemented in the alloys of the last third generation: namely, a decrease in the amount of Li with a higher Cu content to improve ductile characteristics. As a result, two groups of high-strength ultralow-weight high-modulus corrosion-resistant weldable Al-Li-based alloys were formed (Table 5). The first one is typical of alloys containing 1.2–1.8% Li and 2.0–3.0% Cu, and the second one, 0.6–1.3% Li and 3.0–4.5% Cu [13]. For example, to the first group, there belong the alloys 2076, 2099, 2190, 2196, 2297, 2397, and 1461; to the second—the alloys 2050, 2055, 2060, 2065, 2195, 2198, and 1469. Interestingly, comparing the properties of alloys such as 2060, 2199, 1461, and 1469 with the properties of the alloy 2024, widely used in the world practice for aircraft fuselage cladding, one can obviously reveal the advantages of Al-Li-based alloys in strength, ductility, toughness, and fatigue crack resistance over the latter alloy [13]. 

In dependence of the content of alloying elements, at HT and TMT in the alloys one can find the formation of various disperse phases: δ’ (Al_3_Li) and δ (AlLi), θ’ and θ” (Al_2_Cu), T_u_ (Al_7_LiCu_4_), T_1_ (Al_2_LiCu), T_2_ (Al_6_CuLi_3_), S_1_ (Al_2_LiMg), and the dispersoids β’ (Al_3_Zr) and Al_3_Sc [2,3,4].

Finally, fourth, HT and TMT technological processes play an essential role along with doping, which determine the mechanisms of formation of granular, subgrain, and intragrain fine defective structures (first of all, determining the type of decomposition, including those types that are due to homogeneous and heterogeneous decomposition with the precipitation of excess phases) [2,4]. It is these structure–morphological specific features that are decisive in the deformation behavior of aging alloys. For instance, the mechanical properties listed in Table 6 of two binary Al-Li-based master alloys and a number of commercial doped alloys based on them with similar Li concentrations convincingly demonstrate that the main contribution to the significant strengthening of the latter is made by aging with the precipitation of several types of excess nanophases, in contrast to the strengthening due to an only single δ’ phase in binary alloys.

Of course, the main goals of regulating the multicomponent chemical composition and characteristics of the microstructure parameters and phase composition are the desire to provide favorable levels of key physical and mechanical properties of alloys (reduction of density, increase of modulus of elasticity and toughness, fatigue durability, and crack resistance), while leveling out the existing negative characteristics (namely, low ductility and fracture toughness, high anisotropy of the durability properties of semifinished products).

Understanding of the influence of the chemical composition and microstructure on the mechanical and corrosion properties allows simultaneously optimizing the chemical composition of alloys, their HT and TMT, and developing general metallurgical and materials science principles of alloying by various elements of alloys of this system [4,16].

### 3.2. Binary Master Al-Li Alloys

**Figure 1:**
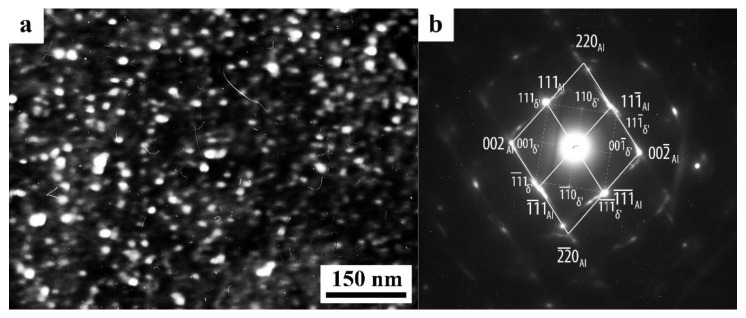
(**a**) The dark-field image of a microstructure of the alloy 1461 after quenching from 530 °C for 15 min and subsequent aging at 160 °C for 32 h, taken in the superstructure reflection (110)_Al3Li_ and (**b**) SAED pattern with superstructure reflections from the δ’ phase, the zone axis is close to ${\left[\bar{1}10\right]}_{\mathrm{Al}}$.

The properties of Al-Li-based alloys are largely determined by the nature of the precipitation of a metastable δ’ phase preceding the formation of the stable δ phase (AlLi) with a structure of *L*1_0_ type (Figure 1) [2,35]. The metastable δ’ phase is isomorphic to the matrix, enriched in Al and atomically ordered according to the type *L*1_2_ (Cu_3_Au). The misfit δ_m_ between the crystal lattices of the α and δ’ phases is small and amounts to 0.08–0.3% [35].

In the Al-Li alloys containing more than 1.34% Li, the δ’ phase is formed in the course of quenching the alloy, in the form of chaotically distributed spherical particles of average diameter *d*_0_ ≈ 3.0–4.0 nm, coherent with the α matrix. Such morphology of the precipitated phase is explained by a small value of elastic stresses, which takes place as the misfit δ_m_ between the crystal lattices of the α- and δ’ phases is relatively small: because of the lattices being isomorphous [36]. The solid solution decomposition already in the course of quenching is due to a high concentration of quenched-in vacancies in the field of existence of the δ’ phase because of the sufficient bounding energy between the atoms of Li and vacancies [37].

The results of numerous studies do not allow us to unambiguously clarify the nature and mechanism of formation of the initial products of decomposition of Al-Li alloys during low-temperature aging. Some authors believe that, by analogy with other aging aluminum alloys, spherical Guinier–Preston zones (GPZs) are formed first [38,39,40], others believe that an intermediate δ’ phase (Al_3_Li) is precipitated immediately from the beginning of the decomposition process [36,41]. The possibility of the formation of the δ’ phase in Al-Li alloys by the spinodal mechanism was also discussed [42]. In any case, it can be noted that the activation energy of the formation of precipitates (GPZ or δ’-phase particles) is quite small. In most studies, it was noted that the metastable intermediate δ’ phase is precipitated homogeneously [36,37]; however, its precipitation by the mechanism of discontinuous decomposition was also experimentally detected in [43].

It is important to emphasize separately that the precipitation of the intermediate δ’ phase in the vicinity of grain boundaries leads to the appearance of precipitates free zones (PFZs) [40]. PFZs are formed as a consequence of the decrease in the concentration of quenching vacancies in these areas due to the sinking of vacancies (and, thus, their disappearance) at the grain boundaries.

When the particles of the equilibrium δ phase are precipitated, zones free of the δ’-phase precipitates also appear around them. Particles of the δ phase have the forms of laths and globules, which is likely to be connected with different mechanisms of their formation. With the appearance of the δ phase, the deterioration of the mechanical and corrosion-resistant properties of Al-Li alloys is usually connected.

### 3.3. Mechanical Properties and Plastic Deformation of Aging Binary Al-Li Alloys

The mechanical characteristics of aged binary Al-Li alloys depend on their content of Li. The increase in Li leads to an increase in the yield strength (σ_0.2_) and strength (σ_u_) of the alloys and a decrease in their elongation (δ) (Table 6) [44]. When the precipitation of an ordered δ’ phase coherent with the α-matrix takes place, the main strengthening factors are (i) the resistance to deformation resulting from the appearance of antiphase boundaries (APB) when cutting the ordered coherent δ’ phase by sliding dislocation and (ii) the friction stress in the δ’-phase particle. The remaining factors are considered insignificant and make up no more than 5% of the values of the mentioned factors to be taken into account [44].

When studying dislocations in deformed aged Al-Li alloys, it has been found [45] that at first, they move in pairs and cut the particles of the δ’ phase. The leading dislocation, when cutting the ordered particle of the δ’ phase, destroys the atomic order along the sliding plane, giving rise to the appearance of APB. The second (trailing) dislocation of the pair restores the order, liquidating APB. After prolonged aging (for more than 10^6^ s at 200 °C), paired dislocations are no longer observed, but only single dislocations bent near large particles of the δ’ phase are visible. Such a dislocation structure indicates a change of the mechanism of the interaction between dislocations and δ’-phase precipitates from the cutting of particles to the Orowan mechanism of the passing-by of dislocations, leaving dislocation loops around the precipitates. The average particle size at which the mechanism change was observed in the Al-3.11% Li alloy was close to 50 nm [45].

Plastic deformation in aged Al-Li alloys with a coherent ordered δ’ phase occurs inhomogeneously and is localized in slip bands. If in this case at the same time there appear channels free of precipitates, where dislocations accumulate and form pileups, then these channels can be places of cracks nucleation. The precipitation of the equilibrium δ phase, which occurs most often along the grain boundaries, provokes the formation of PFZs, in which the deformation can also be localized, especially in the places of triple junctions of grains. Thus, the nature of the decrease in the plasticity of high-alloyed Al-Li alloys aged to maximum strength is primarily associated with the inhomogeneity of deformation, leading to the strong localization of deformation, especially near grain boundaries.

### 3.4. Complex-Alloyed Al-Li-Based Alloys

As was noted above, Cu and Mg are the main alloying elements of commercial Al-Li-based alloys, but Zr and Sc, as well as a number of other alloying elements in various combinations, also serve as inevitable additives in almost all known high-alloyed Al-Li alloys.

**Figure 2:**
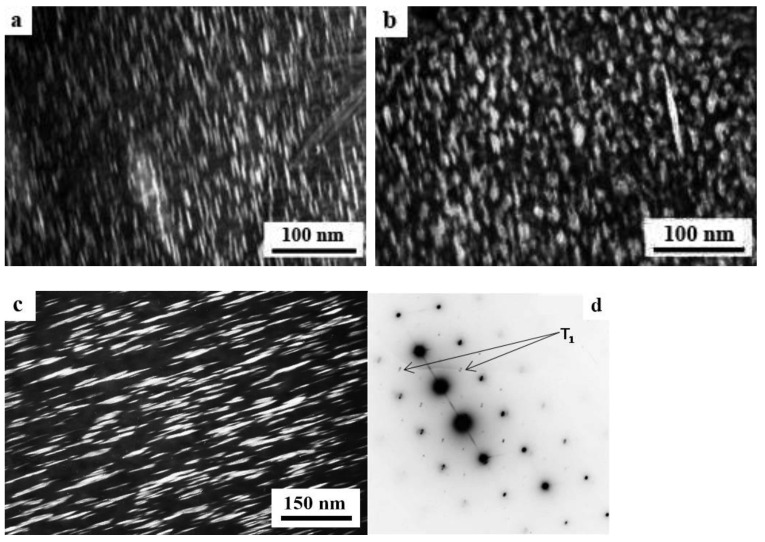
(**a**–**c**) The dark-field images taken in the reflections (**a**) (200)_θ’_, (**b**) (001)_δ’_, and (**c**) (102)_T1_ from the microstructure of the alloys 1450 with additions of (**a**,**b**) Sc and Mg, respectively, and 1469 (**c**,**d**), after quenching and subsequent aging to the maximum strengthening, and (**d**) an SAED pattern with the axis of zone, [110]_Al_.

***Alloys of the system Al–Li–Cu.*** Alloying by Cu of Al-Li alloys, along with the formation of the well-known binary metastable phases θ’ (θ”) (Al_2_Cu) and δ’ (Al_3_Li)—entails the precipitation of the ternary stable T-type phases T_1_ (Al_2_LiCu), T_2_ (Al_6_CuLi_3_), and T_B_ (Al_7_LiCu_4_). The kinetic specific features of the decomposition of Al-Li-Cu solid solutions are characterized by that at a prolonged low-temperature (below 165 °C) aging of the alloys only the metastable precipitates of the phases θ’ and δ’, as well as the T_1_ phase, are formed, while the T_B_ and T_2_ phases do not precipitate [41]. If an alloy contains more than 2% Cu, than the θ phase is always present in the volume of alloy at any content of Li. The appearance of the T_1_ phase in the course of aging of an alloy is always detected at a content of about 0.8% Li, while the formation of the metastable δ’ phase is observed in the alloys with the content of Li greater than 1.2% [41]. The ratio of precipitated volumes of the θ’ and δ’ phases is controlled by the contents of Cu and Li in the alloy. The phases θ’ and δ’ can interact with each other, with forming a new type of two-phase composite particles (see Figure 2a,b) [46]. At increasing the rate of quenching, the activation of the precipitation of the θ’ particles occurs, at interphase faces of which the δ’ phase heterogeneously nucleates, with the formation of composite δ’/θ’ particles [46].

**The T_1_ phase (Al_2_CuLi).** The T_1_ phase, depending on the Li content in the alloy, can be either stable (with Li less than 2%) or metastable (with Li more than 2%). In the latter case, this phase is formed only in the low-temperature field of existence, and the stable T_2_ and δ phases are precipitated at enhanced temperatures [41]. The T_1_ phase nonisomorphous to the matrix has a hexagonal crystal lattice with 12 atoms per unit cell. This phase nucleates preferably in heterogeneous way, just at the defects of the crystal lattice of the matrix, in the form of plates of hexagonal shape with the habitus along the atomic plane {111}_Al_ (Figure 2c). The T_1_ phase contributes to the strengthening of Al-Li-Cu alloys by 2–10 times greater than that from the δ’ phase. Moreover, precipitation of the T_1_ phase is accompanied by increasing the modulus of elasticity of the alloy. The Young modulus of the T_1_ phase accounts to *E* = 350 GPa, which is considerably larger than the value of the δ’ phase (*E* ≈ 97 GPa) [47]. The influence of the T_1_ phase on the ductile characteristics of Al-Li-based alloys is ambiguous. Nucleation of the T_1_ phase along the grain boundaries in the form of continuous film is one of the reasons of a low ductility of these alloys [18,46,48]. The activation of a uniform nucleation of this phase in the bulk of a grain and the decrease in the quantity of precipitates on the boundaries considerably enhance the ductile characteristics of the alloys.

**The T_B_ phase (Al_7_LiCu_4_).** One can expect the appearance of the phase T_B_ in the Cu-enriched alloys with a Li content of less than 1%. Because of the proximity of the structures of the θ’ and T_B_ phases, we assume that when Li is dissolved in the θ’ phase, the θ’ phase in this case continuously passes into the T_B_ phase [41]. However, the presence of the T_B_ phase is detected only after aging at 350 °C. Long-term low-temperature aging in the region of the existence of a metastable θ’ phase does not lead to the appearance of a T_B_ phase [49].

**Figure 3:**
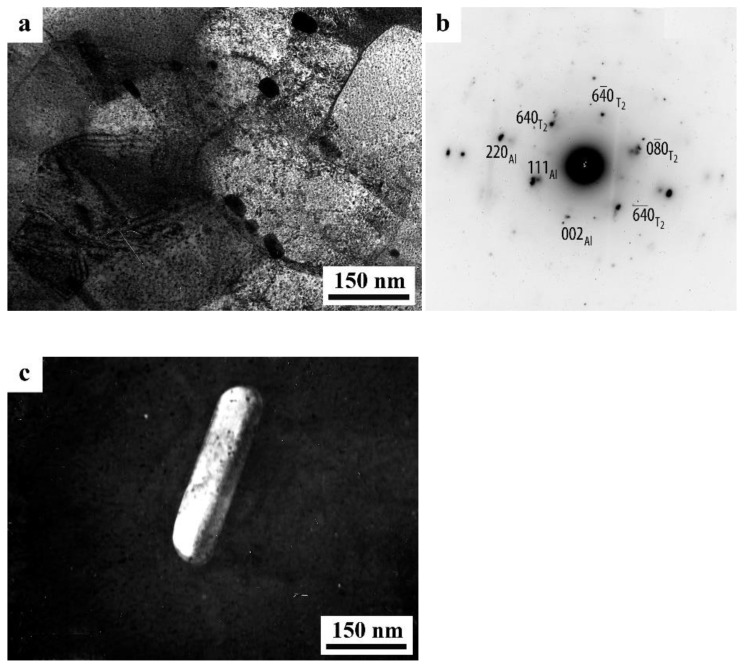
(**a**) Bright- and (**c**) dark-field images (in the reflection (640)_T2_) of the microstructure of the alloy 1461 after quenching from 530 °C, 15 min and subsequent artificial aging at 160 °C, 32 h, and (**b**) SAED pattern with reflections from the T_2_ phase, the zone axis close to [110]_Al_.

**The T_2_ phase (Al_6_CuLi_3_).** In the alloys enriched in Li more than 2%, with a 0.4% Cu content, when aging below 190 °C, the T_2_ phase is formed heterogeneously along the grain boundaries, having the BCC structure with a lattice parameter of *a* = 1.3914 nm (Figure 3) [49]. When the aging temperature rises above 190 °C, then, along with the T_2_ phase, a phase with icosahedral symmetry appears. An increase in the temperature and aging time facilitates the precipitation of the T_2_ phase at both high-angle and low-angle boundaries [50]. The maximum volume fraction of its (T_2_) precipitation was observed after aging at 400 °C, for 24 h. The appearance of the T_2_ phase deteriorates the mechanical properties of Al-Li alloys, since the presence of this phase at the grain boundaries is accompanied by the appearance of zones free of δ’ particles. The T_2_ phase is brittle and, as is shown by fractographic studies, intercrystalline fracture is observed in alloys aged to maximum strength initiated by coalescence of pores arising around particles of the T_2_ phase [50].

**Alloys of the system Al–Li–Mg.** Alloys of the Al-Li-Mg system are the lightest of aluminum ones in specific weight. The addition of Mg to Al-Li alloys causes their additional solid–solution strengthening and an increase in strength characteristics after aging. Thus, according to the equilibrium phase diagrams, Mg initiates a decrease in the solubility of Li and Cu in Al, increasing the supersaturation of alloys, which causes acceleration of the nucleation and growth kinetics of the main hardening phases [48]. The strengthening of an Al-Li-Mg alloy is mainly due to the precipitation of a metastable δ’ phase, as well as of the S_1_ phase (Al_2_LiMg), in it [51,52]. In the alloys doped by 2% Li, the ternary S_1_ phase is formed at a concentration of Mg greater than 2%, and for alloys with 4% Li, at a concentration of Mg greater than 1%. The binary magnesium phases occur at a much higher Mg content. With an increase in Mg content over 2%, an equilibrium S_1_ phase occurs in the form of long coarse rods or laths of up to 2 microns long, growing along <110> directions [52]. The growth of the S_1_ phase is accompanied by the formation of epitaxial dislocation loops at the butt ends of the rods, with providing the relaxing of elastic stresses. In their turn, these loops can be the sites of nucleation of new particles, which also are found to generate loops when the process takes on an autocatalytic character.

**Small-percent additives in Al-Li-based alloys.** Insignificant amounts of Co, Ti, Y, Mn, Ni, Zr, Sc, Cr, Cd, Ge, In, Sn, Zn, Ag, and their combinations, for example, in the form of Al-Ti-B and Ti-C-Al master alloys, are introduced to improve the mechanical properties of many an Al-Li alloy [53,54,55,56,57,58,59,60]. Some of these chemical elements form small, coherent dispersoids with an ordered structure that are not cut by moving dislocations during plastic deformation. The formation of such precipitations, on the one hand, leads to a moderate strengthening; on the other hand, which is much more important, the formation of these particles has a significant effect on the grain size, the stability of the deformation structure, the kinetics of the decomposition of a supersaturated solid solution during aging.

Among the most important factors affecting the structural-phase transformations and properties of alloys, when alloyed with minor additives, the following can be distinguished:

1. the formation of complexes between quenching vacancies and atoms of alloying elements, which prevents the formation of GPZs and, consequently, leads to a decrease in the width of PFZs;
2. an increase in the GPZs solvus line, causing a change in the stability region (or metastability) of the phases;
3. the formation of segregations at the interphase boundary between the nucleus of precipitation and the matrix, thereby facilitating the process of nucleation of phases by lowering the surface energy and contributing to the formation of highly disperse precipitations;
4. the formation of additional strengthening phases;
5. the formation of clusters at the initial stages of the aging process, stimulating heterogeneous nucleation of the main strengthening phases;
6. a decrease in the solubility of the main alloying elements in the aluminum matrix, leading to a higher supersaturation of the solid solution in the quenched state.

The grain size is an important characteristic of Al-Li alloys, which largely determines their mechanical properties and the nature of fracture. First of all, this is related to that Al-Li-based alloys having a coarse-grained structure exhibit the low ductility and fracture toughness in result of the appearance of deformation hardening and the localization of stresses in both cases along grain boundaries. In these cases, the majority of commercial aluminum alloys after various HT and TMT demonstrate the presence of a course-grained recrystallized structure. That is why the refinement of a grain structure by means of additional alloying is a necessary condition of producing modern Al-Li-based alloys and is implemented via the (following) main approaches:

1. Grain refinement of aluminum alloys during crystallization from the melt is usually achieved by introducing up to 0.05% Ti in the form of Al-Ti-B or Al-Ti-C ligatures containing Al_3_Ti, TiB_2_, or TiC compounds. In turn, small additives of Zr in Al-Li-based alloys can reduce the effect of grain refinement, which is offset by adding more ligature (increasing the effective concentration of Ti) or replacing Al-Ti-B with Al-Ti-C [53,60,61,62].

2. The increase of the temperature of recrystallization and the slowing down of the grain growth in Al-Li-based alloys are often achieved by their alloying with transition elements, among which the most common additives are Cr, Mn, Zr, and Sc. The relatively low solubility and diffusion rate of these elements lead to the formation of dispersoids and to a significant slowdown in grain growth at elevated temperatures.

**Figure 4:**
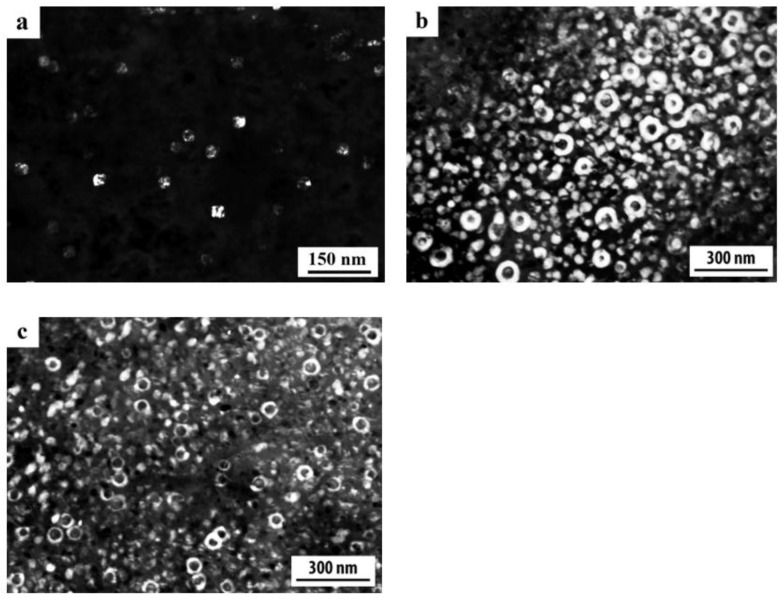
(**a**,**b**) Dark-field images of the microstructure of the alloys (**a**) 1469, (**b**) 1450, and (**c**) 1450 with additives of Sc and Mg after quenching and subsequent aging to maximum strength; the images are taken in the superstructural reflections (110)_Al3(Sc,Zr)_ and (110)_Al3Li_.

**Alloys of the System Al–Li–Zr and Al–Li–Sc.** Zr is an invariable additive to all commercial Al-Li-based alloys, as it serves as an effective modifier of the cast structure and an antirecrystallizer of the deformed alloy and thereby causes significant structural hardening. Already when 0.1...0.2% Zr is added, a coherent metastable β’ phase (Al_3_Zr) is formed with an ordered structure of *L*1_2_ type and a lattice parameter *a* = 0.408 nm (Figure 4a) [4,35]. In numerous literature data, it is noted that in Al-Li alloys, equiaxed particles–dispersoids are formed during high-temperature heating under quenching or during homogenization. With subsequent aging, the δ’ phase nucleates, when—heterogeneously, on the interfacial surfaces of β’ particles, forming composite particles δ’/β’ (Figure 4b) [63,64]. The formation of such particles increases the resistance during plastic deformation to cutting (the dispersoids) by moving dislocations. This is due to the fact that moving dislocations cannot cut the β’ phase particles because of the high AFG energy [57]. In this case, the deformation is carried out by bypassing (via bending of dislocations around) the β’ phase particles, which significantly reduces the localization of stresses and changes the character of the plastic deformation of the alloy from inhomogeneous to more homogeneous. Thus, the Zr additive not only contributes to the efficient refinement of grain during crystallization and increases the recrystallization temperature but also increases the uniformity of plastic deformation.

Sc, having a low solubility in Al (0.2–0.3%), like Zr, is an intensive modifier of the structure of Al-Li alloys [65]. In Al-Sc-based alloys, an Al_3_Sc compound with an ordered *L*1_2_-type structure with a lattice parameter of 0.4105 nm is in equilibrium with an aluminum solid solution. Along with a strong modifying effect, Sc also has an antirecrystallization effect, which exceeds the corresponding effect of the most commonly used antirecrystallizers of Al: Mn, Cr, and Zr [4,35]. Primary Al_3_Sc particles formed during crystallization do not dissolve during subsequent heating under quenching (450, 500 °C). The amount of primary Al_3_Sc particles in cast and hardened Al-Li-Sc alloys depends on the Sc content. The presence of these particles leads to a significant increase in hardness (by 300 MPa) of hardened alloys Al–2.04% Li–0.3% Sc compared with the binary alloy Al–2.04% Li. Doping by Sc leads to a slowdown in the precipitation of the δ’ phase during quenching and at the initial stages of aging, which can be explained by a lower concentration of nonequilibrium quenched-in vacancies. This is due to the presence of effective sinks for them, namely, the interphase boundaries “matrix/primary coherent particles of the Al_3_Sc phase” and a more developed surface of grain boundaries, since significant grain refinement is noted in Al-Li-Sc alloys. Another distinctive feature of the formation of the δ’ phase in ternary Al-Li-Sc alloys is the heterogeneous nucleation of the δ’ phase at the interfaces of the particles of the Al_3_Sc phase with the formation of two-layered particles consisting of the core of the Ai_3_Sc phase and the shell of the δ’ phase (Figure 4b), as this takes place in Al-Li-Zr alloys [65].

**Alloys of the System Al–Li–Mn.** Owing to the relatively low self-cost and increased nonequilibrium solubility, Mn is used as a chemical element that increases both the temperature of the beginning of recrystallization and the strength properties of aluminum alloys due to the formation of intermetallic particles, and is present in almost all modern foreign compositions of Al-Li-based alloys (Table 5). Mn is capable of forming a supersaturated solid solution based on aluminum with a content of up to 4 wt.% Mn depending on the cooling rate during casting and the composition of the alloy [1]. The high degree of the supersaturation of solid solution relative to equilibrium concentrations at heat treatment temperatures provides a significant inclination for the decomposition of the supersaturated solid solution and the formation of disperse precipitates of secondary Mn-containing phases. In the Al-Mn system, the equilibrium phase is Al_6_Mn with a crystal lattice of orthorhombic type [21]. However, in the course of decomposition of a supersaturated solid solution, the formation of Al_6_Mn phase can be preceded by the formation of metastable phases, for example, Al_12_Mn with a BCC lattice [66]. Dispersoids of metastable phases with a quasicrystalline type of structure can be formed in alloys with Mn [67,68].

***Alloys of the System Al–Li–Ag.*** It has been shown in many publications that Ag has a significant effect on the kinetics of phase transformations in aluminum alloys due to the high binding energy of Ag atoms with vacancies [69,70], thereby affecting structural and phase transformations and, accordingly, properties [71]. Thus, for instance, in [72], it was shown that the microalloying of alloys based on the aluminum-copper system by magnesium and silver improves their mechanical properties, since small additives of these elements prevent the coarsening of the phases θ’ and θ (Al_2_Cu) precipitated during the decomposition of a supersaturated solid solution and contribute to the formation of new phases: S’ (Al_2_CuMg) and Ω (Al_2_Cu) (the structural type of the latter phase has not yet been precisely established). According to the literature data, the precipitation of the Ω phase in the alloys of Al-Cu-Mg system with the addition of Ag favorably affects their properties at elevated temperatures [73].

Another example of the effect of alloying by Ag on the structure and properties of the alloys used in aerospace engineering is described in [74]. Thus, it was found that after prolonged natural aging, the Al-Mg alloy with a high magnesium content and 0.2% Ag acquired higher strength and plastic properties due to the suppression of heterogeneous nucleation of β-phase (Al_2_Mg_3_) precipitates in the form of a continuous film along grain boundaries and stabilization of the small-angle boundaries formed during crystallization.

In [75], the independent and the combined effect of Ag and Mg additives on the decomposition processes of supersaturated solid solution in Al-4.01Cu-1.11Li-0.19Zr-0.11Ti alloy was studied and the following facts were established:

1. The atoms of Ag segregate at the interphase boundaries along the planes {111}_T1_/matrix and {100}_θ’_/matrix, thereby lowering the elastic energy arising from the mismatch between the phase lattices and the matrix, which stimulates the nucleation of the T_1_ phase, accelerates the growth of T_1_ and θ’ plates, and slows down the formation of GPZs.
2. The addition of magnesium, on the contrary, leads to the activation of the precipitation of GPZs and θ’ phase at the early stages of aging and to the formation of T_1_ at later stages, due to the segregation of Mg atoms on the steps of T_1_- and θ’-phase particles, lowering the energy of misfit in the directions normal to the habitus planes of these phases.
3. The combined addition of Ag and Mg accelerates the nucleation of the T_1_ phase but slows down its growth.

This is due to two interrelated reasons: (a) due to the strong interaction between Ag and Mg atoms, the independent role of Mg in activating the process of precipitation of the θ’ phase somewhat decreases; and (b) the influence of Ag on the acceleration of the growth of θ’ plates in the presence of Mg also decreases. These processes may be related to the fact that a certain number of bound Ag and Mg atoms can segregate on the protrusions of T_1_ plates along the {111}_α_ planes, replacing Cu in the matrix lattice.

Despite the high solubility of Zn in Al at elevated temperatures (up to 70%), and, accordingly, a great potential for dispersion hardening during aging, alloying with this element of Al-Li-based alloys is carried out primarily to increase corrosion resistance and solid–solution hardening and is an essential prerequisite condition for almost all alloys of the latest generation.

### 3.5. The Effect of External Influences on the Decomposition of a Supersaturated Solid Solution of Al-Li-Based Alloys

Any external influence on a supersaturated or decomposed solid solution can affect the mechanism, kinetics of formation or morphology of precipitates by increasing the concentration of defects in the crystal structure, changes in the internal energy of the alloy, grain size, the state of the intercrystalline and interphase boundaries, the amount, morphology, and distribution of the precipitated phase.

**Moderate plastic deformation**. Numerous studies have revealed a significant effect of plastic deformation on the decomposition processes of supersaturated solid solution, as well as on the properties of aging alloys. In the case of Al-Li-master alloys that exhibit decomposition already under the process of quenching, preliminary plastic deformation causes the interaction of dislocations with particles of the intermediate δ’ phase [2]. In other words, the already partially decomposed solid solution is deformed. As a consequence, it becomes necessary to consider the stability of various products of decomposition with respect to plastic deformation, i.e., to investigate the interaction of individual dislocations and their ensembles with the precipitates of excess phases that arose as a result of aging or formed on a defective substructure after deformation. At the same time, it follows from the data given in [4] that the plastic deformation of binary Al-Li alloys virtually does not affect the mechanism of precipitation of the δ’ phase. However, when studying multicomponent alloys of the Al-Li-Cu-Mg-Zr type, it has been shown that deformation leads to an increase in the strength characteristics of these alloys while maintaining ductility at a good level [76]. The authors associated this phenomenon only with an increase in the volume fraction of the phase S (Al_2_CuMg). In Al-Li-Cu-Zr alloys, the main strengthening factor after three percent deformation was the activation of heterogeneous precipitation of particles of the stable T_1_ phase due to an increase in the number density of dislocations, which are favorable places for the phase nucleation [77]. Activation of the T_1_ phase precipitation in the volume of grains was accompanied, in turn, by a decrease in the distribution density of the metastable phases δ’ and θ’, as well as of the grain-boundary precipitates of the T_1_ phase [77].

**Conditions of quenching.** Other equally important factors affecting the kinetics of the decomposition process are the conditions for obtaining a supersaturated solid solution. By changing the quenching parameters (temperature and cooling rate), and adjusting the degree of supersaturation of the alloy with quenching vacancies, it is possible to influence the (i) kinetics of the decomposition of the supersaturated solution, (ii) morphology, (iii) mechanisms, and (iv) the nature of the nucleation and separation during aging of phases along the boundaries and inside the grains. In alloys experiencing the spinodal or the isomorphic decomposition, especially with a high atomic content of the alloying element (about 10%), it is usually impossible to obtain a single-phase state by quenching due to insufficient cooling rates less than 10^4^ K/s. Therefore, Al-Li alloys already contain δ’-phase particles in the quenched state. Thus, by changing the quenching conditions, it is impossible to completely suppress the nucleation of the δ’-phase particles, but it is possible to change the kinetics and the character of their precipitation during subsequent aging.

The analysis carried out in [77] has revealed that an increase in the quenching rate of Al-2% Li-3% Cu-0.1% Zr alloy leads to the activation of the heterogeneous nucleation of the δ’ phase on the θ’-phase plates and precipitates of T_1_ phase in the grain volume, which results in increasing the strength without changing the values of plasticity parameters. Also of interest are the results of a study of the aging process of an Al-2.5% Li alloy quenched from the liquid state [78]. A high volume fraction of PFZ (zones free of precipitates) was found (60% compared to 4% for conventional quenching), which affected the coalescence of the δ’-phase particles due to their greater number density of distribution in the grain volume. Interestingly that, despite the large extent of the area of the grain boundaries in the alloy due to the significant grain refinement, the δ phase is detected on them only after aging at 240 °C for 36 h, whereas when quenching from a solid state, it is observed at large-angle boundaries almost immediately [78].

### 3.6. Basic Methods for Obtaining Bulk Nanostructured Materials

Very important characteristics of the grain structure of metallic materials are the sizes of grains (subgrains), their size distribution, grain shape, texture and crystallographic orientation, the dominant type of interface and the uniformity of the entire structure, and, consequently, the properties in different sections of the sample. Bulk UFG nanomaterials have a unique structure and, consequently, special properties due to the large extent of the grain boundaries, which opens up prospects for improving existent and creating fundamentally new structural and functional materials [79,80,81]. SPD methods have been widely used for the formation of nanostructures in bulk materials, which make it possible to achieve large plastic deformations of materials at relatively low temperatures without fracturing samples [80]. The HPT method has received great attention and development. For the first time, installations for high-pressure shear deformation in Bridgman anvils were apparently used in research carried out at the Institute of Metal Physics of the Ural Branch of the Russian Academy of Sciences [82,83,84,85]. The other deformation methods for obtaining nano- and submicrocrystalline structures in metals and alloys are the Equal-Channel Angular Pressing (ECAP); Cyclic Extrusion Compression [86,87]; Accumulative Roll Bonding—ARB [88,89]; Repetitive corrugation and straightening [90,91]; Twisted extrusion [92,93]; Differential velocity sideways extrusion—DVSE [94]; Sideways and Forward extrusion [95]; All-round forging [96], and others.

***Evolution of the microstructure of pure metals and alloys at SPD.*** The character of the nanostructure in the course of its formation is determined both by the materials themselves (initial microstructure, chemical and phase composition, type of crystal lattice) and by the SPD conditions (temperature, velocity, method, and degree of deformation, etc.). As a rule, a decrease in temperature, an increase in the applied pressure, an increase in the number of alloying elements contribute to the refinement of the structure and the achievement of the smallest grain size at HPT. At the same time, the nanostructures obtained via SPD represent a complex structural state, which is characterized not only by a small grain size but also by a high level of stresses and distortions of the crystal lattice, the presence of a substructure, different misorientation angles between crystallites, the type of crystallographic texture, and phase composition.

To date, a number of SEM, TEM, and HRTEM studies have established that the evolution of microstructure in various metals in the HPT process occurs in stages [85,97,98,99,100,101]: from the nucleation and development of a cellular structure to a fragmented structure, with the subsequent transition to a partially or completely recrystallized grain structure. The occurrence of diffusion-controlled processes in the highly deformed samples that have undergone recovery and recrystallization [101] at room temperature is apparently possible due to an excessively high concentration of vacancies, exceeding, according to some estimates, the thermodynamically equilibrium concentration by about 15 orders of magnitude [102,103]. According to M. Zehetbauer and his colleagues, one of the most probable reasons for the accumulation of such a high concentration of point defects during deformation is the action of high quasihydrostatic pressures, which lead to lowering the diffusion coefficient of the metal, thereby suppressing the migration of vacancies to their potential sinks [102]. It is possible that when a certain critical concentration of vacancies is reached, the flows of point defects are activated in the direction towards the boundaries, which contributes to the launching of the recrystallization mechanism.

In model alloys subjected to HPT, the evolution of the microstructure has features similar to those of the corresponding process in pure metals. During the deformation of alloys and intermetallides, not only a strong size refinement of the microstructure occurs but also do the changes in the phase and chemical composition associated with the (i) formation of supersaturated solid solutions, (ii) crushing of the second phase particles, and (iii) amorphization possible for some systems [104,105,106,107,108,109,110,111,112,113,114,115,116,117,118,119,120,121,122,123]. The formation of nanostructures in alloys is strongly affected by their phase composition. In single-phase solid solutions, the structure evolves similarly to that which is characteristic of pure metals, but the resulting grain size is usually much smaller [124,125]. In multiphase alloys, the nature and morphology of the second phases play an essential role in the (size) refinement of the structure. If there are particles—of the second phases in the initial structure of the alloy—stronger than the matrix, at HPT, their crushing can occur, as well as dissolution due to mechanical alloying, leading to the formation of a supersaturated solid solution. The SPD-induced behavior of the commercial alloys has been studied much less.

**Resistance of SPD-produced nanostructures to external influences.** The nanostructures obtained by SPD methods are characterized by significant (or average) atomic displacements from the sites of the ideal crystal lattice due to the high number density of defects. Therefore, these materials have high stored energy and are metastable. In this regard, the question of their resistance to external influences, primarily to such as temperature and applied stresses, is of extreme importance. Grain growth in nanostructured materials after SPD, as in other nanomaterials, begins at relatively low temperatures close to 0.4 *T*_melt_ and at the temperatures even lower [126,127,128]. The evolution of the structure during heating of various nanostructured materials has a number of general characteristic features. As a result of the processes of structural recovery (redistribution of dislocations and a decrease in their density in the grain volume and in the nonequilibrium boundaries formed during SPD), a simultaneous decrease in the long-range stress fields and elastic distortions of the crystal lattice occurs. In this case, a polycrystalline structure with small grain sizes is formed. The stage of nucleation is allowed to be virtually absent and usually at this stage there is no migration of grain boundaries, and the mechanism of recrystallization is close to the mechanism of in situ recrystallization. However, if the boundaries of some grains are able to migrate at the expense of neighboring grains, the recrystallization mechanism will be close to the usual process of recrystallization via migration of grain boundaries. If, for some reason (for example, as a result of the appearance of precipitates), the migration of the boundaries of new grains becomes difficult, a recovery may occur mainly, and a structure will form, mainly with smaller but misoriented grains. If individual grains with nonequilibrium boundaries are preserved in the structure after the recovery, an abnormal growth of such grains in the process of ongoing recrystallization is possible.

The specific feature of commercial alloyed multicomponent alloys consists in a gradual SPD-induced phase transition from a multiphase state to a single-phase state of a supersaturated solid solution. In the course of acts of subsequent annealing, a reverse phase transition to a more equilibrium state is realized by precipitation of inclusions and coarsening of excess phases. Since the decomposition of solid solution in Al alloys, as a rule, is ahead of grain growth, this is successfully used to improve the structure and properties of nanostructured materials. These processes have been extensively studied in the course of SPD of aging Al alloys of different systems: Al–Cu [104,105], Al–Cu–Mg [106,107,108], Al–Mg–Si [109,110,111,112], and Al–Zn–Mg [113,114,115,116,117], including also Al–Li [112,118,119,120,121,122]. The temperatures at which certain processes of structure evolution occur depend primarily on the chemical composition, the structure of the material, and the applied SPD methods that determine the features of the deformation substructure.

To date, the mutual influence of these two mechanisms of structure evolution (a change in the defective structure of the crystal lattice and a change in the chemical distribution of atoms of different chemical elements) during SPD and subsequent annealing of deformed structures remain insufficiently studied for multicomponent Al-Li-based alloys. Undoubtedly, the study of their mutual influence, as well as the study of the relationship between structural changes and changes in properties, has an important place in further research aimed both at understanding the fundamental processes occurring during the annealing of multiphase alloys subjected to SPD, and at studying the thermal stability of submicrocrystalline materials in their commercial application.

### 3.7. The Structure of the SPD Alloys in the Nanostructured State

Analysis of the structure of the HPT alloys showed that their structural state was determined first of all by the chemical composition and by the value of deformation controlled by the number of revolutions *n*.

**Figure 5:**
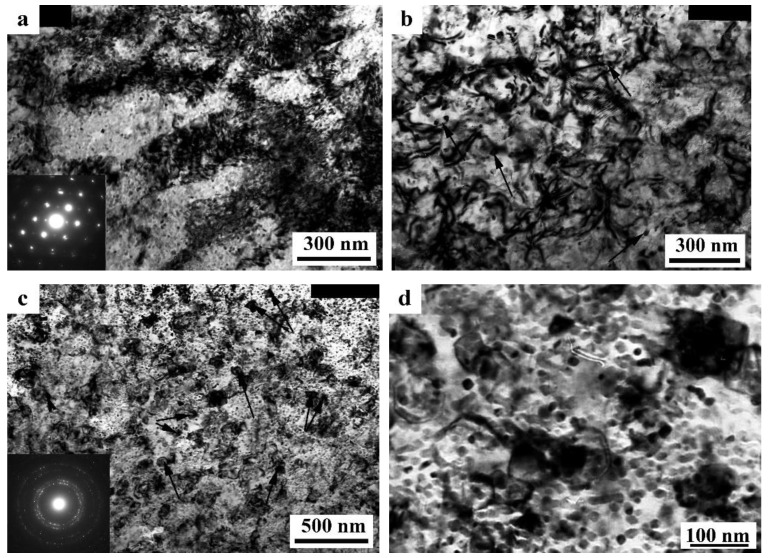
Bright-field images of the microstructure and the corresponding SAED patterns of the 1450 alloy with additives of Sc and Mg after HPT; (**a**)—*n* = 0.25; (**b**)—*n* = 5 (note dislocation loops); (**c**,**d**)—*n* = 10.

At a moderate value of deformation with *n* = 0.25 revs, in the alloy 1450, a developed cell structure has formed (Figure 5a). The boundaries of cells have presented by themselves wide tangles of dislocations; the diameter of the central parts of cells characteristic of a lowered number density of dislocations has varied from 0.3 to 1 µm. A somewhat smeared shape of the reflections in the corresponding electron diffraction patterns has pointed out at a low-angle crystallographic misorientation between the cells. In this case, the presence of the δ’ nano phase was recorded in the SAED pattern (insert in Figure 5a) [129,130].

An increase in the degree of HPT deformation always led to the disappearance of the dispersoids Al_3_(Zr, Sc) and to the formation of an equiaxed UFG structure characteristic of a grain diameter of 100–150 nm, with/of different, including high-angle, misorientation (Figure 5b,c). The UFG fragments were preferably separated by the boundaries of dense tangles of dislocations. Along a number of boundaries of the UFG fragments, the formation of large-scale so-called dipole boundaries took place just inside the fragments (Figure 5b). For the first time, such fragments were observed in the works of Rybin [131]. According to the studies [131], the defects of this kind are formed during SPD and they entail approximately the same—but oppositely directed—rotations (of value greater than 1°) of adjacent (neighboring) areas of material.

During the SPD process (or immediately after it, during natural aging), the supersaturated solid solution decomposes to form a stable T_2_ phase. Nanoparticles with a diameter of 5–10 nm were precipitated mainly heterogeneously, at the boundaries of fragments, including dipole boundaries. It is worth noting that in high-strength Al-Li fine-grained (FG) alloys the T_2_ phase is formed only after overaging of the alloys. Similar results were found earlier in [132] on the UFG alloy 1450, in which the phase composition changed during artificial aging: disperse particles of stable phases T_1_ and T_2_ were precipitated along the grain boundaries and in the volume of grains, and the formation of a metastable phase δ’ in the volume of grains and the nucleation of extended T1-phase precipitations along their boundaries were suppressed.

The increase in the degree of deformation ensured the formation of a more disperse and uniformly fragmented UFG structure: both the average sizes of the fragments and nanograins and their size spread were found to decrease. Fragmentation of the grain structure was accompanied by an increase in the length of the dipole boundaries, their number, and improvement of the boundaries of nanofragments—their narrowing occurred (Figure 5b). The most fragmented and homogeneous structure was formed after the maximum deformation produced after a rotation to *n* = 10 rev. In addition, perfect equiaxed nanograins with reduced number of internal defects were found to be commensurate with deformation-produced UFG nanofragments (Figure 5c,d). Their observation indicates the course of dynamic recrystallization at HPT. The simultaneous development of the relaxation and recrystallization processes during SPD deformation also contributed to a decrease in the density of dipole boundaries [129,130].

The images of UFG fragments show a characteristic heterogeneous structure: either evenly distributed specific contrast (shown in Figure 5) was detected in their volume, or areas with a thickness of 10–15 nm separated by narrow low-contrast borders (Figure 5). The detected electron diffraction contrast effects make one suggest that secondary weakly misoriented nanoregions are formed inside the primary UFG fragments and nanograins. The secondary nanofragments are separated by low-angle boundaries and probably arise as a result of the “cyclic” stage-by-stage nature of the SPD process. At the same time, in the dynamically recrystallized grains formed under the action of continuing SPD at HPT, the accumulation and rearrangement of defects such as dislocations and disclinations occurs again, and the process of nanofragmentation repeats [133]. These effects are studied in more detail in [134]. The increase in deformation leads to (i) a slight increase in the size of nanofragments and to (ii) misorientation between them. The change in the deformation mode with an increase in the number of anvil revolutions did not have a noticeable effect on the volume fraction and the nature of the distribution of the T_2_ phase. Particles of high dispersity (with a diameter of no more than 10 nm) were well visualized at the boundaries of nanograins and at the preserved fragments of dipole boundaries.

**Figure 6:**
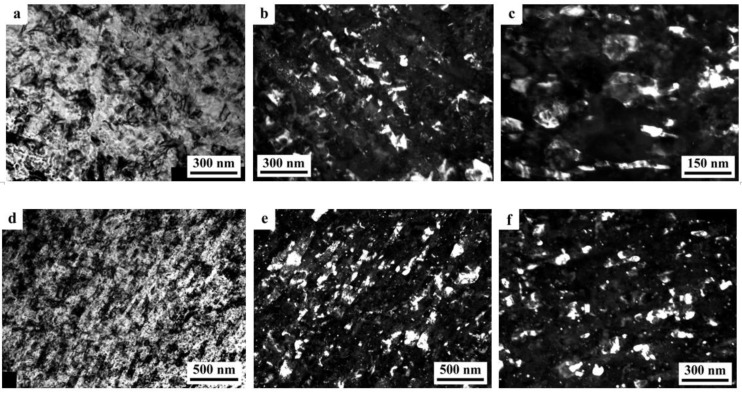
The (**a**,**d**) bright- and (**b**,**c**,**e**,**f**) dark-field images of the microstructure of the alloy 1461 after HPT, taken in the (442)_T2_ and (111)_Al_ reflections; (**a**–**c**)—*n* = 5; (**d**–**f**)—*n* = 10.

The SPD of the samples of alloys 1461 and 1469, using HPT, led to similar structural changes in their structure, namely, to the formation of a UFG state with a typical predominantly nanofragmented structure, against which dipole boundaries were revealed [135,136]. An increase in the degree of HPT deformation was accompanied by fragmentation of the structure and the development of dynamic recrystallization. The latter process manifested itself in an increase in the proportion of recrystallized nanograins commensurate with fragments, and in a decrease in the length and density of dipole boundaries or their almost complete disappearance (Figure 6). At the same time, the deformation structures of alloys 1461 and 1469 had a number of features and differences.

A characteristic feature of the alloy 1461 after HPT deformation at a large number of revolutions *n* was the formation of a pronounced banded structure. Thus, after HPT, *n* = 5, this weakly pronounced banded contrast with a characteristic band width of 50–100 nm was detected in some parts (of TEM images) of the sample (Figure 6a–c). Further HPT deformation, *n* = 10, led to an increase in the intensity of the extended banded contrast, and deformation bands up to 100 nm wide were well visualized throughout the entire volume of the sample, in the volume of which dislocation-free nanograins with a diameter of up to 50 nm were detected (Figure 6d–f) [135].

**Figure 7:**
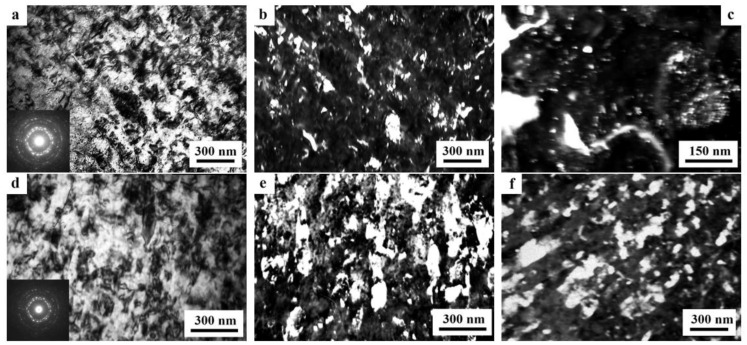
The (**a**,**d**) bright- and (**b**,**c**,**e**,**f**) dark-field images of the microstructure of the alloy 1469 after HPT, taken in the (442)_T2_ and (111)_Al_ reflections; (**a**–**c**)—*n* = 5; (**d**–**f**)—*n* = 10.

A decrease in the number of alloying elements and additional alloying with Ag in the alloy 1469 initiated acceleration of structural and phase transformations after deformation compared to the alloys 1450 and 1461 [136]. Already after HPT deformation of *n* = 1, a nanofragmented structure was observed, at the background of whose image the individual recrystallized nanoparticles with a diameter of 20–50 nm were revealed (Figure 7). A similar structure in the alloy 1450 was formed after HPT deformation of *n* = 5. The change in the chemical composition of the alloy caused the activation of the decomposition processes of the supersaturated solid solution with intensive heterogeneous precipitation of the stable phase T_2_ at the boundaries of nanofragments and dipole boundaries. The simultaneous development of the processes of deformation-induced fragmentation, dynamic recrystallization, and precipitation of the second phases contributed to the formation of the most disperse structure among all the studied alloys and at maximum deformation HPT, *n* = 10, and led to the formation of a recrystallized structure, which was accompanied by the complete disappearance of dipole boundaries. The increase in the degree of deformation slightly intensified the decomposition of the supersaturated solid solution: there was a slight increase mainly in the number of nanosized particles of the T_2_ phase.

**Figure 8:**
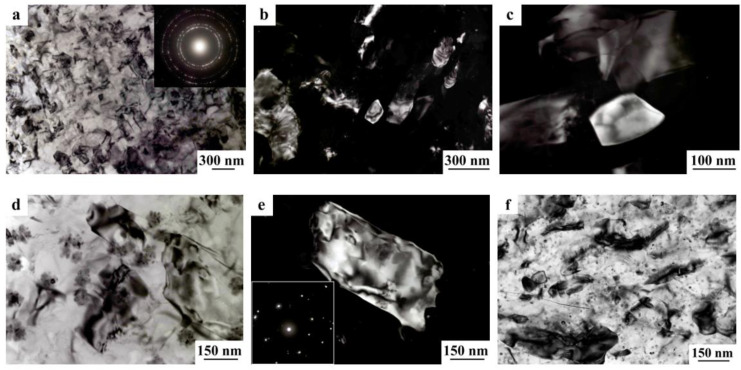
The (**a**,**d**,**f**) bright- and (**b**,**c**,**e**) dark-field images of the microstructure of the alloy 1441 after HPT, taken in the reflections (111)_Al_, (200)_Al_, (530)_T2_; inserts of (**a**) ring- and (**e**) pointlike SAED patterns (zone axis [001]_Al_).

A distinctive feature of the structure of HPT alloy 1441 was the formation of a much more equilibrium partially recrystallized structure. So, after HPT, *n* = 5, a mixed UFG structure was observed in it: SMC structure with an average diameter of grains ~0.3–0.4 microns and NC one, with a grain diameter of less than 100 nm (Figure 8a,b). The crystal grains had an equilibrium or close to such shape, straightened boundaries and were characterized by a large-angle misorientation relative to each other. The significant development of dynamic recrystallization and the formation of a more equilibrium UFG structure led to the complete disappearance of dipole boundaries and a decrease in the number of other defects.

Against the background (in the images) of a homogeneous UFG structure, areas consisting of fragments of deformation nanostripes were identified (Figure 8d,e), along the boundaries of which SMC and NC regions were nucleated (Figure 8e). The stripes were divided into regions with a low-angle misorientation with a diameter comparable with the size of nanograins of 70–100 nm, which was confirmed by the observation of inhomogeneous contrast and framework of low-contrast boundaries visualized in certain reflective positions. The analysis of the blurring of matrix reflections on the corresponding SAED patterns showed that the azimuthal misorientation of these regions was insignificant and ranged from 30 to 60 arc min (Figure 8f).

The bright- and dark-field images of the HPT alloy structure revealed an insignificant number of the highly disperse phases S_1_ and T_2_ with a diameter of no more than 10 nm, heterogeneously generated at the grain boundaries. In addition, (the images of) ultradisperse (with a diameter of less than 1 nm) δ’-phase particles were detected on bright- and dark-field images of the alloy, which were taken in matrix reflections of (002)_Al_ type (Figure 8e).

Thus, the stages of SPD processes in high-strength Al-Li-Cu alloys were established and the evolution of the alloys microstructure was demonstrated: from a cellular structure characteristic of moderate deformation to fragmented and banded ones with an increase in the degree of SPD deformation to partially or completely recrystallized structures, formed as a result of the dynamic crystallization induced by SPD. The most characteristic structural features are revealed and changes in such specific defects of the crystal structure as dipole boundaries are traced, depending on the magnitude of deformation and relaxation of stresses accumulated during SPD. The observed dependence of the length of the dipole boundaries on the amount of deformation allows us to conclude that the formation of dipole boundaries is not only the result of SPD itself but also of a relaxation mechanism of the energy stored at SPD. In accordance with this, it is obvious that an increase in their length with an increase in the number of revolutions is the result of an increase in stored energy. The transition of the alloy to a more equilibrium state as a result of partial dynamic recrystallization at maximum deformation (by *n* = 10 revs) leads to a decrease in the length of the dipole boundaries and their density. In other words, there is a hierarchical change in the mechanism of relaxation of stored stresses and lattice distortions according to defective structural states.

The formation of a highly defective structure in the SPD alloys is accompanied by an intensification of the decomposition of a supersaturated solid solution and leads to a change in the phase composition of the alloys: whereas the metastable δ’ and stable T_1_ phases are precipitated in the samples with an FG structure during artificial aging, the formation of a high-temperature T_2_ nanophase occurs in the samples with a UFG structure at room temperature.

### 3.8. Annealing of SPD Alloys

**Figure 9:**
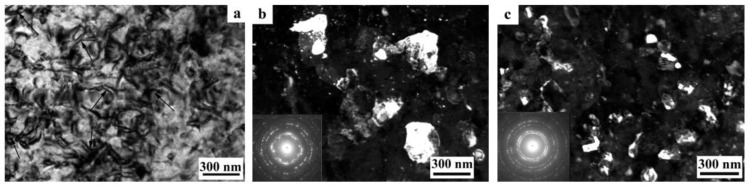
The (**a**) bright- and (**b**,**c**) dark-field images of microstructure of the alloy 1450 after its HPT at (**a**,**b**)—*n* = 1, (**c**)—*n* = 10 and annealing at 150 °C for 15 h; images taken in the reflections (111)_Al_ and (200)_Al_, with the (**b**,**c**) corresponding SAED ringwise patterns.

It has been established that during the annealing of SPD-processed alloys, static recrystallization and decomposition of a supersaturated solid solution occur simultaneously, in result of which a recrystallized structure of various scale was formed [129,137,138]. The nanograins formed during recrystallization had predominantly equiaxed shape or shape close to it, well-formed boundaries and were characterized mainly by large-angle misorientation (Figure 9). The transition of SPD alloys during annealing to a more equilibrium state also led to the complete disappearance of the initial dipole boundaries, which contributed to the relaxation of the elastic stresses accumulated during SPD. At the same time, despite the general similarity of the structural changes occurring during annealing, the specific structure in the alloys was largely determined by the annealing mode, structural features of the deformed state, and chemical composition, i.e., this specific structure exhibited phase-structural heredity.

**Figure 10:**
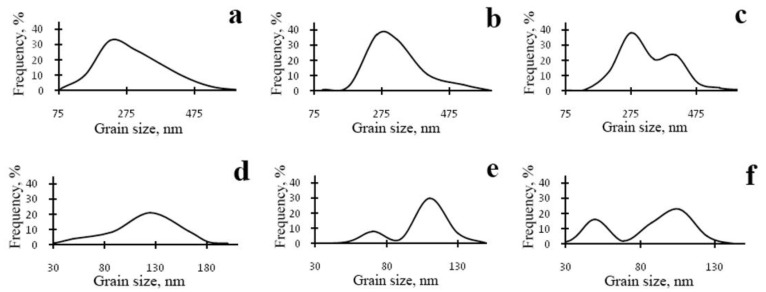
Frequency dependences of the grain size distribution in the alloy 1450 with the addition of Sc and Mg after HPT and subsequent annealing, (**a**–**c**)—190 °C 10 h: (**a**)—*n* = 1, (**b**)—*n* = 5, (**c**)—*n* = 10; (**d**–**f**)—150 °C 15 h: (**d**)—*n* = 1, (**e**)—*n* = 5, (**f**)—*n* = 10.

So, annealing of alloy-1450 samples after HPT led to the formation of a recrystallized UFG structure in the alloy. After annealing at 190 °C, 10 h, depending on the deformation conditions, the diameter of equiaxed grains with high-angle boundaries varied from 200 to 300 nm (Figure 10) [129]. To preserve the NC grain structure in the alloys 1461 and 1469, the annealing time at 190 °C was reduced to 30 min, but even such a short annealing of SPD alloys did not allow to stabilize the NC structure. Therefore, in the future, annealing at a lower temperature of 150 °C was used to obtain nanosize-scaled grains.

This led to the formation of the mixed SMC and NC structures in the alloys (Figure 10). The increase in the dispersion of the grain structure can be associated both with a known slowdown in the kinetics of recrystallization with a decrease in the annealing temperature, and with the features of the decomposition of a supersaturated solid solution having realized during annealing and having a barrier effect on grain growth.

The character of the boundaries of recrystallized nanograins was determined primarily by the amount of deformation. So, in the case of HPT, *n* = 1, nanograins often had nonequilibrium convex–concave boundaries with increased energy (Figure 9a), which is especially characteristic of the alloy 1450. The number of such crystallites consistently decreased with increasing deformation. After HPT, *n* = 10, the nanograins with straightened low-energy boundaries became dominant (Figure 9b) [129].

The nonequilibrium state of convex–concave boundaries was confirmed by the observation of diffraction contrast effects in the form of loops and arcs on light-field images near them (the image of such loops is shown in Figure 9c) due to the presence of elastic stress fields and dislocations. It is known that in materials with an NC structure dislocations are “pushed” out (expelled) from the volume of nanograins to their boundaries due to the so-called “image forces”, where these dislocations are fixed and further become potential sources of another dislocations when interacting with elastic internal and external stress fields.

The existence of nanograins in the annealed alloys with different types of boundaries (nonequilibrium convex–concave and more equilibrium rectilinear) indicated that different stages of recrystallization were realized for different structural elements during low-temperature annealing: postprimary and postprimary recrystallization. Nanofragments formed during the SPD process underwent primary recrystallization, during which nanograins with convex–concave boundaries were formed, and dynamically recrystallized nanograins experienced postprimary recrystallization, which led to the improvement of their shape and (straightening) of the boundaries. In accordance with the fact that the intensity of dynamic recrystallization increased with increasing deformation, during annealing after HPT, *n* = 1, primary recrystallization prevailed, and after HPT, *n* = 10, postprimary recrystallization began to be realized for most of the grains.

The secondary nanofragments formed during SPD were preserved in the volume of nanograins even after low-temperature annealing. These nanofragments were observed both in nanograins formed by transformation of primary nanofragments, and in nanograins formed earlier in the process of dynamic recrystallization. At the same time, annealing contributed to a certain increase in secondary nanofragments diameter to 20–30 nm and the angle of misorientation between them. The analysis of SAED patterns from clusters of several (3–5) nanofragments allowed us to estimate the azimuthal component of misorientation (AM) of nanofragments: under the SPD modes used, AM is ranged from 30 arc min to 2–3°.

In the annealed alloy 1450, the bimodal grain size distribution was most clearly revealed (Figure 10). Thus, on the histogram of grain size distribution for the sample after HPT, *n* = 10, 2 maxima were observed corresponding to the average grain diameter of 50 and 100 nm; lowering HPT to *n* = 5 led to the formation of a more uniform grain size structure with a predominance of grains with a diameter of 100–110 nm. The minimal degree of deformation (*n* = 1) was characterized by the almost complete disappearance of the bimodal distribution. At the same time, the bimodal grain size distribution was maintained also for the samples annealed at 190 °C after the HPT at *n* = 10. One can assume that the appearance of grain-size bimodality is also caused by the simultaneous realization of the mechanisms of the primary and the postprimary recrystallization during annealing.

**Figure 11:**
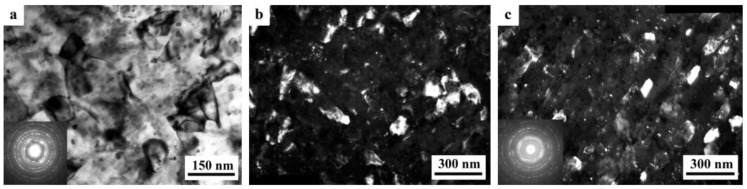
The (**a**) bright- and (**b**,**c**) dark-field images of microstructure of the alloy 1461 after its HPT at (**a**)—*n* = 1, (**b**)—*n* = 5, (**c**)—*n* = 10 and subsequent annealing at 150 °C for 15 h; images were taken in the reflections (111)_Al_ and (200)_Al_, with the (**a**,**c**) corresponding SAED ring-wise patterns.

The samples of alloy 1461 subjected to HPT at 5 and 10 revolutions, after annealing, partially inherited the banded [or stripewise] structure formed during deformation [137]. After HPT, *n* = 5, in the structure along with a predominantly homogeneous grain distribution, separate areas with a banded structure were identified, in which the NC- and SMC grains were distributed anisotropically along crystallographic directions that coincided with the directions of deformation bands, forming extended conglomerates of grains with a length of more than 200 nm (Figure 11). The diameter of the NC grains was 50–70 nm, and the diameter of the SMC grains was 150–170 nm (Figure 11a–c). The banded structure formed over the entire volume of the sample after HPT, *n* = 10, was inherited after annealing (Figure 11c). The subsequent heat treatment caused the annealed samples to widen these deformation bands–stripes from 50–100 nm to 100–200 nm (Figure 11a,b). The presence of a banded structure led to an anisotropic distribution of the SMC- and NC grains and to a change in their shape: they, like their clusters, were observed to have stretched along the crystallographic directions of the deformation bands (Figure 6). This trend was most noticeable in nanograins, for which the ratio of width to length was approximately 1 to 2.

**Figure 12:**
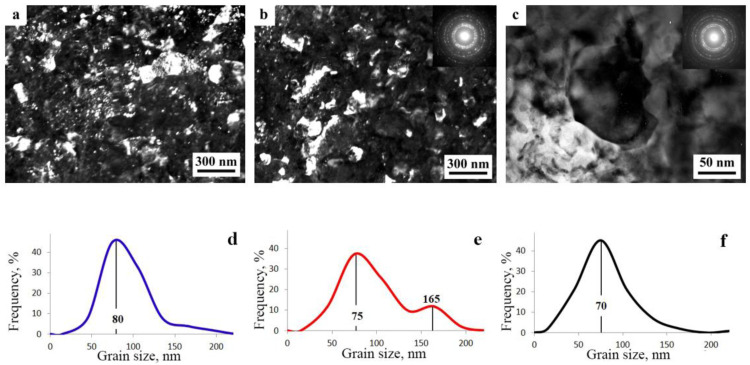
(**a**,**b**) Dark-field and (**c**) bright-field microscopic images, (**a**,**b**) taken in reflections (111)_Al_, (200)_Al_; and (**b**,**c**) the corresponding ring-wise SAED patterns and (**d**–**f**) frequency curves of grain-size distribution, all characteristic of the alloy 1469 after HPT and subsequent annealing at 150 °C, for 15 h: (**a**,**d**)—*n* = 1, (**b**,**e**)—*n* = 5, (**c**,**f**)—*n* = 10.

Annealing of the alloy 1469 led to the formation of the most disperse NC structure in it among all the alloys studied (Figure 12). So, after HPT, *n* = 1, and annealing, the sizes of the nanograins mainly varied in the range of 30–100 nm; after HPT and annealing, *n* = 5, —40–80 nm; and after HPT and annealing, *n* = 10, —30–50 nm (Figure 12d–f) [138]. The formation of a maximally size-dispersed NC structure during annealing in the alloy 1469 was apparently associated with the intense deformation-induced precipitation of T_2_-phase particles in the SPD process along the boundaries of nanofragments and dynamically recrystallized nanograins due to additional alloying of the alloy by Ag. Numerous studies have noted the tendency of the silver and magnesium atoms to interact with excess vacancies and with each other, thereby changing the kinetics of the decomposition of supersaturated solid solution during aging and increasing the strength characteristics of these alloys [139]. The interaction of vacancies with Ag atoms in solid solution was indirectly indicated by the absence of dislocation vacancy loops in the annealed structure, despite the fact that, according to [140], recrystallization processes in NC materials are usually accompanied by a sharp increase in the number of excess vacancies and often the appearance of loops.

Annealing of the alloy 1469 did not cause a noticeable change in the sizes and size distribution of the precipitates of T_2_ phase, with the exception of particles homogeneously nucleated in the body of the SMC and NC grains (Figure 12). The sizes of such particles did not exceed several nanometers and their volume fraction remained insignificant. In its turn, an increase in the SPD preceding annealing (an increase in the number of revolutions of HPT from 1 to 10) just contributed to an increase in the total number density of the T_2_ phase with a slight increase in the sizes of particles of T_2_ phase from 5 to 15 nm. So, in the alloy 1469 subjected to HPT and subsequent low-temperature annealing, the particles of the stable T_2_ phase retained a high degree of the size dispersion and had an effective barrier effect on the growth of nanograins during annealing, which ensured the stabilization of the predominantly NC structure.

Additional alloying of the alloy 1450 by Mg led to the precipitation of the S_1_ (Al_2_LiMg)-phase particles in the form of flat disks along with the T_2_ phase. In the alloy aged at 150 °C, disperse particles of the T_2_ phase were precipitated mainly at the boundaries of the nanograins, and particles of the S_1_ phase—both at the boundaries and in the volume of the nanograins (Figure 9). The increase in the annealing temperature to 190 °C was accompanied by coagulation of the T_2_- and S_1_-phase particles and by a significant decrease in their number density [129].

**Figure 13:**
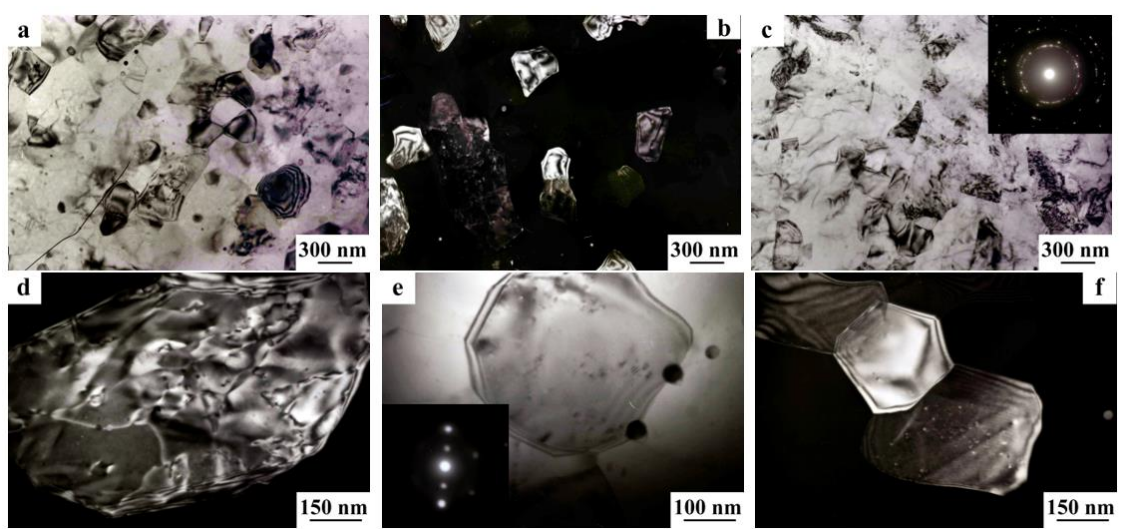
(**a**,**c**,**e**) Bright- and (**b**,**d**,**f**) dark-field microscopic images of the structure, taken in the reflections (200)_Al_ of SAED patterns from the alloy 1441 after HPT, *n* = 5, and subsequent annealing at 160 °C, for 15h; (**c**,**e**) the corresponding electron diffraction patterns: (**c**) ring-wise and (**e**) nodewise, the latter with zone axis [011]_Al_.

After annealing at 160 °C, 15 h, a homogeneous recrystallized structure was observed in the alloy 1441 subjected to the HPT, *n* = 5 (Figure 13)—the structure being morphologically similar in many respects to the traditional FG structure after recrystallization annealing. The grains had an equiaxed shape, a clear cut, straightened boundaries, and equilibrium triple junctions (Figure 13b) and were free from dislocation-type crystal lattice defects. The average diameter of such grains ranged from 0.3 to 0.5 microns. In some parts of the sample after annealing, fragments of a banded structure formed during HPT with low-contrast boundaries of bands–stripes were preserved (Figure 13b). Within such bands, grains of elongated or irregular shape were detected, the length of which could exceed 1 micron. Dense pileups of dislocations were observed in their volume (Figure 13f).

Rarely located equiaxed particles of the S_1_ and T_2_ phases were detected at the grain boundaries of the annealed alloy (Figure 13e). The diameter of the grain-boundary precipitates after annealing increased to 40 nm, but the number density of the particles virtually did not change. This suggests that for the initial state that existed, this HT regime corresponded, by analogy with FG alloys, to the conditions of overaging, and in the course of annealing, the supersaturated solid solution experienced additional decomposition with subsequent coagulation of precipitates. In addition to grain-boundary precipitations of stable phases, the disperse precipitates of δ’ phase with a diameter of up to 10 nm were also detected in the volume of grains (Figure 13f).

Thus, the formation of the UFG structure in alloys significantly affected the phase transformations in the studied alloys and caused a change in the sequence of decomposition of the supersaturated solid solution during aging with the formation of stable highly disperse T_2_- and S_1_ phases which in the studied alloys with the FG structure are precipitated in the form of extended plates only during high-temperature annealing (overaging modes) at temperatures more than 250 °C. According to [79], the influence of SPD on the character of phase transformations can be caused by changes in the thermodynamic conditions of phase equilibrium due to the high number density of high-energy interface surfaces (boundaries of nanofragments and nanograins) and to an increase in the number of defects in the crystal lattice. The presence of these factors contributes to the formation of phases that do not exist in the alloy in the usual MC state. In this case, high-temperature stable phases are precipitated at room temperature; their morphology and degree of dispersion change.

On the other hand, the precipitation of highly disperse particles had a significant barrier effect on the growth of nanograins during annealing and led to the fixation and stabilization of the NC state. From a comparison of the microstructures of the alloys 1450, 1469, and 1461, which are characterized by both the precipitation of a large number of highly disperse particles of stable T_2_ phase and the stabilization of the NC state—from a comparison with the case of the alloy 1441 (namely, its structure), characterized by an SMC-grain structure with rarely located particles of the phases S_1_, T_2_, and δ’, it is clearly seen that the barrier effect is provided primarily due to the heterogeneous precipitation of stable T_2_-phase particles. Thus, despite the high degree of alloying of the alloy 1441 (Table 2), the lower Cu content in it, compared to other alloys studied, did not allow obtaining a sufficient volume fraction of the copper-containing phase T_2_ and fixing the NC state.

### 3.9. The Effect of Natural Aging on the Structure and Properties of the SPD Alloys

Studying of the effect of long-term storage on the stability of the UFG structure and corresponding properties is an urgent task, since for all structural materials, the storage (i.e., natural aging) at ambient temperature is an inevitable stage in the technological chain of the manufacturing, processing, storage, and exploitation. In the case of storage, natural aging is often accompanied by structural changes in the alloys and leads to deterioration of their properties.

**Changes during natural aging in the grain structure of the preliminary SPD alloys.** Electron microscopic analysis showed that significant changes occurred in the structure of the studied alloys after natural aging. It was found that the fragmented structure of deformation origin formed during HPT was unstable and, during storage (natural aging), transformed into a partially recrystallized NC- and SMC structure with reduced quantity of defects; this transformation was accompanied by the decomposition of a supersaturated solid solution with the precipitation of highly disperse equilibrium phases. The completeness of the recrystallization processes during natural aging was determined by the features of the initial deformed structure (or by the amount of deformation) and by the duration of subsequent storage at room temperature.

**Figure 14:**
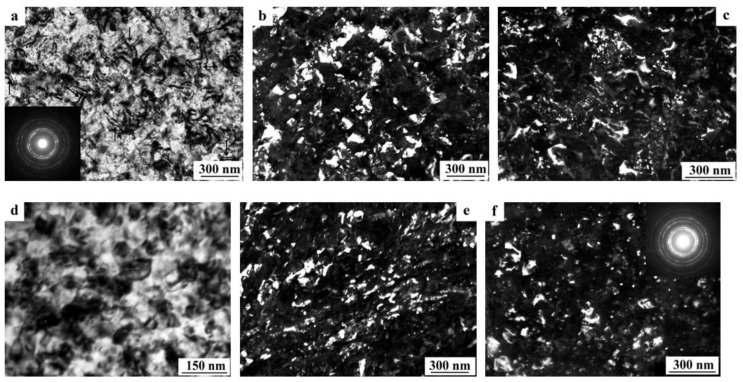
(**a**,**d**) Bright- and (**b**,**c**,**e**,**f**) dark-field images, taken in reflections (111)_Al_, (200)_Al_ and (**a**,**f**) the corresponding SAED patterns, from the microstructure of the alloy 1461 after HPT at (**a**–**c**)—*n* = 1; (**d**–**f**)—*n* = 5; and its subsequent storage for (**a**,**b**,**d**,**e**) 1 month and (**c**,**f**) 1 year.

It was established that when the alloys 1469 and 1461 deformed by HPT at *n* = 1 were then stored for 1 month [141,142], as a result of static recrystallization, a certain transformation of the deformed structure occurs, in result of which the proportion of recrystallized nanograins with an average size of 30–70 nm increased (cf. Figure 14). Grains, unlike fragments, were characterized by a homogeneous contrast, a more equiaxed shape, and more perfect borders with large angles of misorientation. Some of the formed NC- and SMC grains had straightened boundaries, and some had less equilibrium convex–concave ones (Figure 14c).

The ongoing processes of static recrystallization led to relaxation of the elastic energy accumulated during deformation, which was accompanied by a gradual decrease in the number density and extent of the dipole boundaries. However, the preservation of a large number of fragments and sectional fragments of dipole boundaries indicated that there was no complete relaxation of the stresses accumulated during deformation.

An increase in the HPT revolutions to *n* = 5 led to an acceleration of the recrystallization kinetics and the formation of a more equilibrium grain–subgrain structure consisting of fragments and grains, with a predominance of nanograins with a diameter of 30–100 nm. Nevertheless, during the considered period of storage, the process of static recrystallization remained incomplete, which was confirmed by both the presence of dipole boundaries and the nonequilibrium boundaries of the formed nanograins. It can be assumed that the nanograins of a rounded shape without a clear cut with convex boundaries were transformed from nanofragments already during static in situ recrystallization, and that ones with flatter boundaries were transformed by further development of static recrystallization due to straightening of grain boundaries that previously appeared in the course of SPD in the process of dynamic recrystallization.

The nonequilibrium state of the convex–concave boundaries of nanograins after natural aging of an alloy, as well as after annealing, was confirmed on bright-field TEM images by the presence of diffraction contrast effects in the form of single and double vacancy loops near these boundaries (Figure 14).

The appearance of deformation bands in some parts of the sample was a characteristic feature of the microstructure of alloys after HPT, *n* = 5, especially in the alloy 1461. After storage, the tendency to pronounced banding increased, which was clearly revealed in the dark-field images of the alloys (Figure 14b,e). One of the results of the formation of areas with an inhomogeneous unidirectional band substructure consisting of “bands” up to 100 nm thick was anisotropy in the distribution of nanofragments and nanograins along noncrystallographic directions corresponding to the prevailing direction of rotational mass transfer at SPD [142].

**Figure 15:**
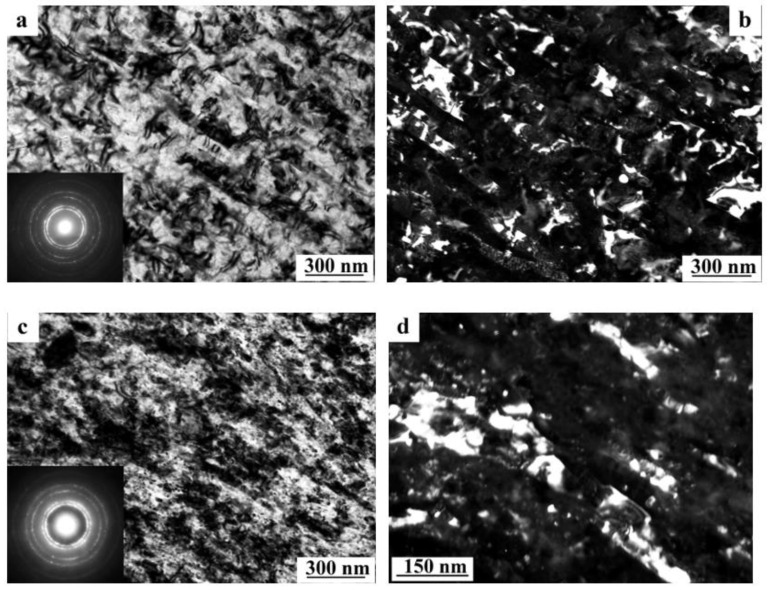
(**a**,**c**) The bright- and (**b**,**d**) dark-field images of the microstructure, taken in the reflections (111)_Al_, (200)_Al_, and (**a**,**c**) the corresponding SAED patterns, of the alloy 1461 after HPT, *n* = 10, and its subsequent storage for (**a**,**b**) 1 month and (**c**,**d**) 1 year.

After natural aging for one month, a structure of the alloy 1461 subjected to HPT, *n* = 10 was characteristic of a developed banded substructure, which consisted of nanograins with a diameter of up to 100 nm; the grains were anisotropically distributed within the limits of noncrystallographic bands of deformation origin (Figure 15). In contrast to the samples deformed by 1 and 5 turns in Bridgman anvils, some coalescence of individual NC grains and secondary nanofragments occurred during the period of short-term storage (i.e., natural aging).

**Figure 16:**
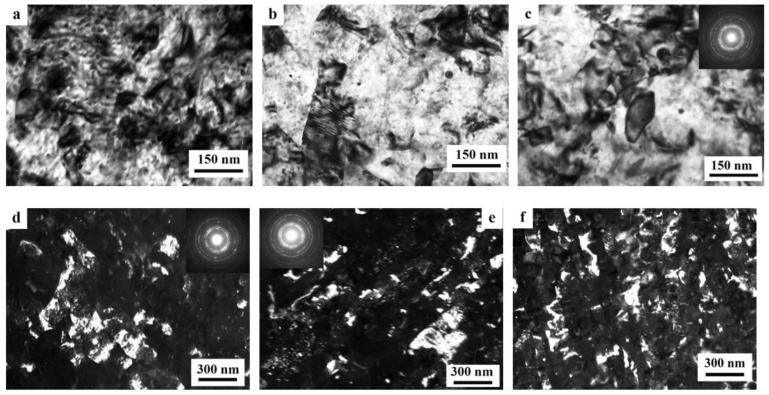
(**a**–**c**) The bright- and (**d**–**f**) dark-field microstructure images, taken in the reflections (111)_Al_, (200)_Al_, and (**c**–**e**) the corresponding SAED patterns from the alloy 1469 after the its HPT at (**a**,**d**) *n* = 1; (**b**,**e**) *n* = 5; (**c**,**f**) *n* = 10 and storage (natural aging) for 2 years.

It was previously shown that at HPT to *n* = 10, a structure close to recrystallized with two types of grains was formed in the alloy 1469: (1) equiaxed NC grains up to 50 nm in diameter and (2) larger elongated SMC grains up to 200 nm in size. Further storage up to 3.5 years did not have a noticeable effect on the state of the grain structure and on the character of nucleation and the volume fraction of excess phases, with the exception of homogeneous precipitation of T_2_-phase particles. In this case, and even after a more prolonged storage, the structure continued to remain nonequilibrium, which was confirmed by the preservation of diffraction effects such as loops and arcs near the boundaries of the nanograins (see Figure 16).

**Figure 17:**
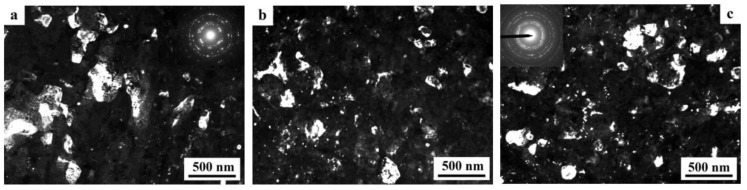
(**a**–**c**) The dark-field images of recrystallized grains taken in the reflection (200)_Al_ and (**a**,**c**) the ring SAED patterns from the alloy 1450 after the HPT and subsequent natural aging at 20 °C, for 1.5 years: (**a**)—*n* = 0.5; (**b**)—*n* = 5; (**c**)—*n* = 10.

In the most alloyed alloy 1450, structural changes proceeded more slowly [143,144]; however, even in this case, after 1.5 years of storage, the formed NC and SMC grains were visualized on the microstructure images. It should be particularly noted that in the samples of the alloy 1450 deformed by the smallest number of revolutions *n* = 0.25, the initial misoriented cellular substructure also turned into a mixed NC + SMC grain structure. The characteristics of the grain–subgrain structure formed during storage were largely determined by the regime of SPD (Figure 17). Thus, after SPD at a pressure of 4 GPa, the dispersity and uniformity of the grain structure increased from the number of revolutions ranged from 0.25 to 5 (see Table 7). After SPD to the maximum value (of deformation), the dispersity in the grain size increased, up to the formation of a bimodal grain size distribution.

At the same time, unlike the annealed alloy, in the alloy after the SPD and subsequent natural aging, most of the grains formed had predominantly nonequilibrium convex–concave boundaries, and only a small part of grains had flatter relatively equilibrium boundaries. The fraction of the latter increased slightly with an increase in the degree of SPD (Figure 17).

Grain boundary dislocations forming walls or short dipoles were detected at the boundaries of individual nanograins, which distinguished them from more extended and curved dipole boundaries when examined visually. The formation of grain boundary dislocations in the form of flat walls or dipoles is quite reasonable, since they are the most stable dislocation configurations in highly nonequilibrium nanocrystalline materials [140,145,146].

The nonequilibrium state of the boundaries of SMC- and NC grains and the preservation of short fragments of dipole boundaries along the interfaces indicated the absence of complete relaxation of stored energy during the natural aging of the alloy under study after SPD and after 1.5 years of storage at room temperature. During storage, the length and density of the distribution of dipole boundaries decreased sequentially with an increase in the number of revolutions from 0.5 to 5, and in the samples after HPT at *n* = 10, the dipole boundaries almost completely disappeared.

In contrast to the alloy 1450, an increase in the duration of storage at room temperature for more than a year of the alloys 1469 and 1461 led to a noticeable development of the static recrystallization process with the formation of a more equilibrium structure represented by recrystallized NC- and SMC grains (cf. Figure 14, Figure 15, Figure 16 and Figure 17).

In the structure of the alloy 1461 subjected to HPT at *n* = 1, after 1 year of storage, regions of fragmented structures of different scales continued to coexist. The incompleteness of the recrystallization process was additionally confirmed by the high size dispersion of NC- and SMC grains and the preservation of a certain number of disjointed dipole boundaries and grains with nonequilibrium convex–concave boundaries [142].

An increase in HPT revolutions to *n* = 5 was accompanied by the development of static recrystallization and led to the formation of a more homogeneous structure, mainly consisting of nanograins with a diameter of ~ 50–100 nm, and the disappearance of dipole boundaries. The NC boundaries retained a nonequilibrium convex–concave shape and were distributed anisotropically within the deformation bands up to 100 nm wide (Figure 14). The evolution of the grain structure led to the degradation of the banded substructure, and it began to partially “crumble” during aging (cf. Figure 14 e,f).

An increase in the number of revolutions of HPT to *n* = 10 caused the “spread–scattering” of deformation bands after a duration of up to 1 year; there was a decrease in the length of the borders of deformation bands and a loss of their straightness (Figure 15). The change in the morphology of the bands is allowed to have been a consequence of their segmentation during recrystallization. In favor of this suggestion, we had the fact of disappearance of the contrast from parallel bending contours in the modified bands (Figure 15d). At the same time, the structure previously formed in the process of fragmentation and dynamic recrystallization at SPD basically retained its nanoscale level. The analysis revealed the presence of two types of morphology of nanograins: the size of those of one type reached 100–150 nm, the size of those that belong to the second was commensurate with the size of secondary nanofragments and was 30–50 nm (Figure 15d). The larger grains, of 100–150 nm in size, were most likely formed as a result of dynamic recrystallization during SPD and subsequent coalescence. The NC grains with a size of 30–50 nm could be formed via the transformation of secondary nanofragments by increasing the angles of their misorientation. At the same time, large NC- and SMC grains had predominantly rectilinear boundaries, while smaller grains had convex-concave boundaries.

An increase in the time of storage to 2 years of the alloy 1469 after HPT at *n* = 1 led to the formation of an inhomogeneous bandlike structure consisting of bands about 150 nm thick [141]. The elements of the alloy structure had a bimodal size distribution: there were equiaxed grains and subgrains of 30–70 nm in size (Figure 16) and larger elongated ones of 100–150 nm in size (Figure 16). The images show regions of NC grains alternating with larger SMC grains elongated in one of the general directions; these grains are forming stripes (see Figure 16). One can suppose that the anisotropy of the shape of these grains is due to special mechanism of the recrystallization via “merging” the initial nanofragments with small-angle misorientation (i.e., coalescence of subgrains), which are located along the primary deformation bands.

With an increase in the degree of deformation at HPT of *n* = 5, the process of static recrystallization and the formation of a more stable structure occurred. Thus, the “stripes” became clearer, and their borders were “decorated” with disperse nanoparticles. All grains were characterized by a clearer cut (Figure 16b,e) and the presence of high-angle boundaries (Figure 16). In the case under consideration, a prolonged storage did not lead to a noticeable increase in the size of most grains of the α matrix, the sizes of which remained in the range of 50–100 nm.

The most significant difference between the structures at the early and later stages of natural aging was the change in the internal defect structure of fragments and grains; during storage, there was an increase in the size of secondary nanofragments and the angle of misorientation between them [134]. This was confirmed both by the blurring of diffraction maxima on the SAED patterns, and by an increase in the intensity of specific speckle contrast and images of small-angle boundaries in the grain volume (Figure 16). At the same time, the diameter of individual secondary nanofragments reached 30 nm.

Thus, as a result of long-term storage in the studied alloys deformed by HPT at 1 and 5 revolutions, mixed-type structures were formed, which largely retained morphological signs of the deformed state but also acquired characteristics of a more equilibrium partially recrystallized structure. In this case, the type of a particular structure at the initial stage of natural aging was largely determined by the previous deformation structure (i.e., the amount of deformation), and at later stages the structural state stabilized. So, after a long-term storage of up to 3.5 years, on the one hand, the structure continued to mainly maintain the nanoscale character, and, on the other hand, it remained as nonequilibrium, preserving the features of the deformed state, for example, “striplike pattern” (see Figure 16a).

The samples deformed to the maximum number of revolutions *n* = 10 were characterized by a more equilibrium structure formed largely as a result of dynamic recrystallization at SPD, and, therefore, their structures underwent much less changes during storage. The main changes in the microstructure were associated with the evolution of the sizes and angles of misorientation of secondary fragments and the improvement of the shape of grain boundaries, their straightening, with the preservation of the nanoscale level.

**The processes of decomposition in the SPD alloy under storage.** During storage for 1 month, natural aging of the highly deformed alloy 1450 occurred, the phase composition of which was determined by the previous SPD regime. Thus, after HPT at *n* = 0.25, equiaxial particles of phases Al_3_(Zr, Sc), with a diameter of 10–20 nm and particles of metastable phases θ’ and δ’ uniformly distributed in the grain volume, with a diameter of less than 1 nm were present inside dislocation cells, as well as an insignificant number of composite particles β’/δ’ and Al_3_(Zr*_x_*,Sc_1–*x*_)/δ’, which corresponded to the phase composition of conventional FG alloys after artificial aging [144].

**Figure 18:**
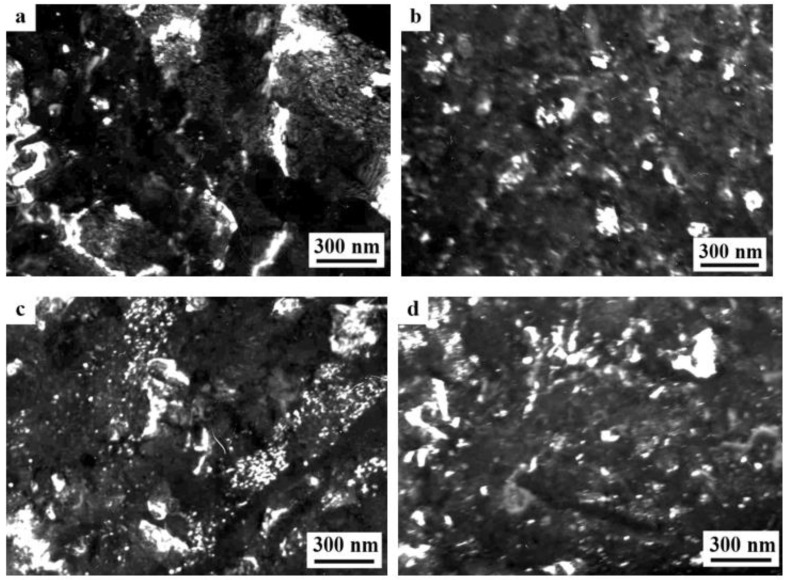
(**a**–**d**) Dark-field images of microstructure of the 1450 alloy after the HPT and storage, taken in (**a**) the reflections (530)_T2_ and (660)_S1_, at *n* = 5, for 1 month; taken in (**b**) the reflection (521)_T2_, at *n* = 0.5, for 1.5 year; taken in (**c**) the reflection (311)_S1_, at *n* = 5, for 1.5 year; taken in (**d**) the reflections (530)_T2_ and (660)_S1_, at *n* = 10, for 1.5 year.

The storage of alloy 1450 samples after higher deformations at HPT (*n* = 0.5–5) led to the formation of stable phases T_2_ and S_1_ (see Figure 18). At this stage of aging, the particles of the two phases were precipitated both heterogeneously, namely, at the boundaries of the nanograins and the preserved dipole boundaries in the form of flat disks with a diameter of about 10 nm (Figure 18), and homogeneously, namely, in the volume of nanograins, as it was evidenced by the weak contrast of the “ripple” type in the images of the NC- and SMC grains.

The intensive heterogeneous precipitation of excess phases at the boundaries of nanograins in the initial stage of natural aging after SPD can be explained by 2 reasons; first, the boundaries of the NC- and SMC grains are the preferred sites of the nucleation of equilibrium phases, and second, the concentration of dissolved matter at the boundaries and in the near-boundaries areas of the grains can be noticeably higher than in their central parts [80]. Thus, the review [147] presents experimental data on the presence of a concentration gradient in nanocrystalline materials, which decreases sequentially from the boundaries of nanograins to the center of each grain. The change in the SPD regime within the specified limits did not have a noticeable effect on the character of the nucleation and growth of the precipitated phases.

An increase in the duration of natural aging to 1.5 year led to a certain increase in the value of volume fraction of the phases T_2_ and S_1_. The increase in the value of previous SPD contributed to (i) a decrease in the number density and (ii) an increase in the size of the precipitates that had nucleated at the boundaries and in the volume of nanograins. So, if after *n* = 0.5, the diameter of the particles of the T_2_ phase did not exceed 10 nm, and that of the S1 phase, 5 nm, then an increase in the number of revolutions to 10 led to an increase in the diameter of the particles of each phase by 2 times (Figure 18).

The performed analysis made it possible to establish that the character of the nucleation and the volume fraction of the T_2_ phase, formed after SPD in the form of disperse particles along the boundaries of the fragments and grains in the alloys 1461 and 1469, virtually did not change during the storage of alloys for 1 month, and the particles of the T_2_ phase remained in the form of disperse precipitates with a diameter of 5–10 nm [141,142]. For the alloy 1469, attention is drawn to the appearance of a diffraction contrast of the ripple type, typical of the occurrence of intragrain mechanism of the formation of the δ’ phase in these alloys. However, superstructural reflections from the δ’ phase were not detected on SAED patterns in the case under discussion. At the later stages of natural aging, confirmation of the formation of the δ’ phase and the corresponding diffraction effects were not found [141].

Prolonged storage for more than a year stimulated heterogeneous decomposition of the supersaturated solid solution: the T_2_ phase was actively nucleated not only at the boundaries of the SMC and NC grains but also in their body at the boundaries of secondary nanofragments. These boundaries, like the boundaries of nanograins, were the preferred places for heterogeneous nucleation of the equilibrium phase T_2_, which led to an increase in its volume fraction (Figure 14b,c). However, the diameter of the particles of T_2_ phase virtually did not change during the period of storage; their diameter still did not exceed 10–15 nm, but the number of the particles increased (see Figure 16e,f).

**Figure 19:**
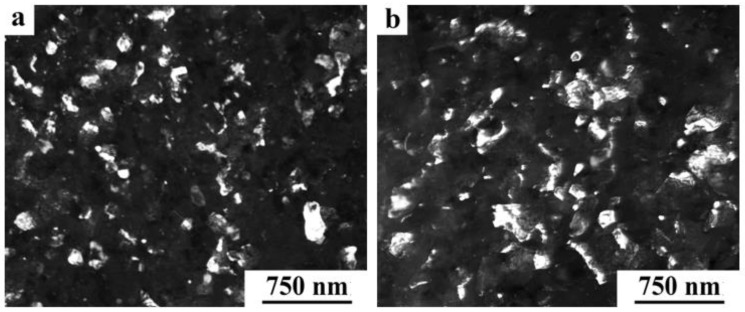
(**a**,**b**) Dark-field images taken in the reflections (111)_Al_, (200)_Al_ from the 1450 alloy after HPT at *n* = 10 and subsequent annealing at 150 °C for 15 h, (**a**) without and (**b**) its after storage for 1.5 year.

***The structure of a highly deformed alloy after low-temperature annealing and storage.*** Prolonged storage for more than three years virtually did not change the diameter and size uniformity of the recrystallized SMC and NC grains of annealed alloys, which was confirmed by comparing dark-field images of the grain structure before and after storage (Figure 19) and analyzing the grain size distribution frequency curves.

After storage, the structure of the alloys still retained the individual nanograins with non-equilibrium boundaries formed during annealing. The preservation of the non-equilibrium boundaries and nano-sized grains in the alloy indicates that primary recrystallization occurred during annealing, and further natural aging did not lead to the transition to the next stage—postprimary recrystallization. Subsequent storage virtually did not affect the size distribution and the volume fraction of stable phases that precipitated during annealing. These phases were uniformly distributed in the volume of grains and along grain boundaries in the form of highly disperse particles.

Thus, low-temperature annealing at 150 °C, for 15 h, used after SPD, provided a sufficiently stable nanostructured and nano-phase state of multicomponent Al-Li-Cu alloys, due to the barrier effect of highly disperse stable nanophases that prevent the growth of nanograins in the alloy after SPD.

### 3.10. The Properties of the SPD Alloys

Using the example of the alloys 1461 and 1469, consider the change in their properties during SPD and subsequent annealing. The performed research showed that the use of SPD ensured the high-strength condition of the studied alloys. The formation of a nanostructured state led to an increase in the microhardness of up to 55%, as well as the reduced elastic modulus *E* by 10–15% compared to the values typical of the alloys with an MC structure processed for the attainment of the maximum strength and hardness (Table 8) [135,136]. In this case, the average plasticity decreased by 6–7%.

The increase in the number of revolutions at HPT (from 1 to 5), as was shown by the results of measuring mechanical properties, provided at first a strong hardening of the alloys, and after 10 revolutions, on the contrary, some softening. The increase in the hardness characteristics was accompanied by an increase in the reduced elastic modulus *E*. Its change also depended on the mode of SPD. For example, for the alloy 1461, the largest increase in the modulus (i.e., by 20%) was realized at *n* = 1, the smallest (by 7%)—at *n* = 5 (Table 8).

Based on the fact that the HPT mode does not have a noticeable effect on the nucleation and growth of the T_2_ phase, we associated the nonmonotonic change in the characteristics of strength, plasticity, and elastic modulus depending on the applied deformation only with the features of the nanostructural state of the alloys under study. Thus, an alloy with a nanofragmented structure (occurred at *n* = 1) had the highest values of plasticity, as well as reduced hardness. The transition from a nanofragmented structure to a mixed one (nanofragmented and nanocrystalline) during partial dynamic recrystallization has provided maximum hardening (*n* = 5). The mixed substructure was nonequilibrium and it partially retained elastic distortions that increased with increasing SPD. Moreover, an additional hardening factor could be the direct refinement of structural elements—nanofragments and nanograins. The presence of a nonequilibrium mixed substructure also caused a natural decrease in ductility. The increase in the size and misorientation of the secondary nanofragments formed in the volume of nanograins could also make a certain contribution to increasing the strength and reducing the ductility characteristics of the alloy.

Activation of dynamic recrystallization in the alloy deformed by 10 revolutions, which is an effective way of relaxation of elastic stresses, led to the formation of a dominant recrystallized microstructure and to some softening of the alloy, accompanied by an increase in plasticity compared with only the nanofragmented state. However, at the same time, the process of decomposition of a supersaturated solid solution was initiated with the precipitation of disperse stable phases, which, on the contrary, should slightly increase strength and reduce plasticity. Thus, the relatively moderate dependence of mechanical properties on the SPD regime was the result of the combined effect of these structural-phase transformations on the mechanical properties of the alloy.

The smaller values of strength gain and decrease in plasticity in the alloys 1469 and 1461 after SPD, compared with the more alloyed alloy 1450, we associate with the development of dynamic recrystallization and a smaller contribution to the hardening from the heterogeneous decomposition of solid solution, which obviously leads to a relative decrease in strength and increase in ductility characteristics.

The lowest strength characteristics were found in the case of alloy 1461, despite its higher degree of alloying by Li and Mg compared to the alloy 1469 (see Table 1 and Table 2). This can be explained by the fact that in the alloy 1469, the Ag additive, due to its high coupling with vacancies, initiates the structural and phase transformations: accelerates the transformation of the nanofragmented structure into a nanocrystalline one and provides an increase in the volume fraction of the strengthening phase T_2_, which has a barrier effect on grain growth and contributes to the formation of a more disperse grain structure.

After annealing, the strongly deformed alloys retained their high-strength state, their hardness characteristics underwent a slight decrease, accompanied by an increase (or preservation at the same level) of plasticity and a nonmonotonic change in the modulus of elasticity (Table 9) [137,138]. The samples annealed after SPD at *n* = 1 had the lowest hardness values, the alloys after SPD at *n* = 5 had the highest hardness. At the same time, the change in the SPD mode virtually did not affect the plasticity of the annealed alloy.

The preservation after low-temperature annealing of the strength and ductility characteristics at the level of the strongly deformed state is due, first, to the size equivalence of the nanofragments and nanograins formed during SPD, on the one side, and the nanograins preserved during subsequent annealing, on the other. Second, annealing did not cause significant changes in the volume fraction of the stable phase T_2_, its size, shape, localization, and distribution density, that is, those parameters that can have, among other things, an adverse effect on mechanical properties.

The decrease in microhardness is associated—in turn—with the relaxation of elastic stresses as a result of the transformation of the nanofragmented, mixed or nanocrystalline structure formed during SPD (depending on the mode or/and regime). The largest drop in microhardness was observed in the alloy 1450, caused by the transformation of the nanofragmented structure into a homogeneous nanocrystalline structure with some SMC grains.

Note that aluminum-lithium alloys differ from other aluminum structural alloys by a higher modulus of elasticity, which significantly expands the scope of their use [148]. Therefore, it is particularly important that the formation of nanofragmented, NC-, or mixed NC- and SMC structures in the alloy 1469 using SPD and, especially, SPD with subsequent annealing, contributes to a further increase in this characteristic.

***Mechanical properties of the 1450 alloy after SPD and storage.*** The results of measuring the mechanical properties showed that the storage of alloy 1450 for 1.5 years after SPD makes it possible to obtain a combination of microhardness of 2.55–2.8 GPa and plasticity of 82% of the alloy similar to that typical of the annealed state.

In this case, the value of microhardness increased slightly from 2.6 to 2.8 GPa with an increase in the number of revolutions from 1 to 5. This was due both to an increase in the density of the interface as a result of an increase in the dispersion of the recrystallized NC and SMC grains (Figure 17), and to the activation of the precipitation of particles of the hardening phases T_2_ and S_1_ (Figure 18).

A slight increase in plasticity during the natural aging of the alloy after SPD is associated with the transformation of the nanofragmented structure into a more equilibrium nanocrystalline one. The absence—in turn—of dependence of plasticity on the SPD regime is due to the following: during recrystallization in the case of SPD at *n* = 5, a stronger relaxation of elastic stresses occurs than that in the case of *n* = 1, which should lead to an increase in plasticity. However, in the case under consideration, the processes that cause a decrease in this characteristic are more actively implemented: a more dispersed structure is formed and a more intensive precipitation of the strengthening phases T_2_ and S_1_ occurs.

***Properties of a highly deformed alloy after low-temperature annealing and storage.*** Measurements of the mechanical properties (MP) of the annealed alloy after its storage showed that they (MP) retained their stability for a long (at least 1.5 year) storage at room temperature, which is in good agreement with structural studies that revealed the dimensional (size) stability of the recrystallized NC structure, the preservation of the degree of the dispersion, the character and density of the distribution of the hardening equilibrium phases that had precipitated during annealing at artificial aging [143].

## 4. Summary and Conclusions

In the present work superlightweight high-modulus commercial Al-Li-based aging alloys of the last generation were selected to analyze the effect of multicomponent alloying and aging on the structure, phase composition, physical and mechanical properties. As a separate one, there was posed and solved the problem of the detailed study of the effect of the SPD by means of HPT on the UFG-structures’ creation and their evolution, including the phase transformations and properties after subsequent thermal treatments. From the analysis of the obtained results taking into account the literature data, the following conclusions could be drawn.

1. Numerous concrete examples of successful long-term flight exploitation of aviation using the multicomponent commercial Al-Li-based aging alloys confirm the potential for their practical use.
2. Al-Li-based aging wrought and weldable alloys of the second and third generations are characterized by favorable levels of key physical and mechanical characteristics (low density, high modulus of elasticity and stiffness, strength, fatigue life, crack resistance, ductility, and fracture toughness).
3. The main strengthening of alloys provides the aging with the precipitation of several types of excess nanophases, in contrast to the strengthening due to a single-acting δ’ phase in binary Al-Li alloys.
4. SPD by means of HPT provides deformation-induced dissolution of dispersoids in all the studied alloys and forms a UFG structure with nano-sized grains with high-angle boundaries. With an increase in the degree of SPD (with the number of revolutions up to 10), along with the fragmentation of the grain structure, dynamic nanorecrystallization was detected, which is an effective way of (i) relaxation of elastic stresses and (ii) some increase in plasticity while maintaining high strength properties of alloys. Also, already in the SPD process or immediately after it, with natural aging, mainly heterogeneous decomposition occurs in alloys with the formation, as a rule, of equiaxial nanoparticles of stable phases.
5. Prolonged storage (1–3 years) at ambient temperature in UFG alloys after SPD causes some change in the substructure of the alloys due to partial headbanding of the structure during static recrystallization in situ and natural aging with the precipitation of stable nanophases.
6. Low-temperature annealing of SPD alloys leads to simultaneous static recrystallization and decomposition of a supersaturated solid solution with the formation of a hierarchical recrystallized UFG structure stabilized mainly by heterogeneous precipitation of equiaxed nanoparticles of stable intermetallic phases.

## Figures and Tables

**Table 1:** Average composition of the studied alloys, wt.%.

Alloy	Cu	Li	Zr	Mg	Sc	Ag	Zn	Al
1441	1.6	1.7	0.09	0.7	0.09	–	–	the rest
1450	3.1	2.0	0.1	–	–	–	–	the rest
1450 modified	3.1	2.0	0.1	0.96	0.08	–	–	the rest
1461	2.8	1.8	0.08	0.5	0.09	–	0.66	the rest
1469	3.2	1.2	0.09	0.3	0.11	0.4	–	the rest

**Table 2:** Average composition of the studied alloys, at.%.

Alloy	Cu	Li	Zr	Mg	Sc	Ag	Zn	The Summary Amount of Alloying Elements, Σ_all.el._
1441	0.65	6.36	0.03	0.75	0.05	–	–	7.83
1450	1.27	7.48	0.03	–	–	–	–	8.78
1450 modified	1.27	7.48	0.03	1.02	0.05	–	–	9.84
1461	1.15	6.78	0.02	0.54	0.05	–	0.26	8.81
1469	1.34	4.61	0.03	0.33	0.07	0.1	–	6.47

**Table 3:** HPT regimes for the alloy1450.

**Pressure *P*, Gpa**	4	4	4	4	8
**Angle of revolution, π rad**	0.5	1	2	10	20
**Number of revolutions *n***	0.25	0.5	1	5	10

**Table 4:** HPT regimes for the alloys 1469 and 1461.

**Pressure *P*, Gpa**	4	4	4
**Angle of revolution *φ*, π rad**	2	10	20
**Number of revolutions *n***	1	5	10

**Table 5:** Average content of alloying elements in Al-Li-based alloys [2,4].

Alloy	Content of Constituents, wt.%								Date of Development, Developer	The Main Strengthening Phases
	Li	Cu	Mg	Zr	Sc	Mn	Ag	Zn (Cd)		
First generation										
2020	1.2	4.5	–	–	–	0.5	–	0.2 Cd	1958, Alcoa	δ’, T_1_, θ’
VAD23	1.2	5.3	–	–	–	0.6		0.2 Cd	1960, VIAM	δ’, T_1_, θ’
1420	2.1	–	5.2	0.11	–	–	–	–	1964, VIAM	δ’
Second generation (Li ≥ 2%)									1980s	
2090	2.1	2.7	–	0.11	–	–	–	–	Alcoa	δ’, θ’, T_1_
2091	2.0	2.0	1.3	0.11	–	–	–	–	Pechiney	δ’, T_1_, S’
8090	2.4	1.2	0.8	0.11	0.17	–	–	–	EAA	δ’, T_1_, S’
8091	2.6	1.9	0.9	0.12	–	–	–	–	EAA	δ’, T_1_, S’
Navalite	2.2	1.2	2.8	0.14	–	–	–	–	Lockheed	δ’, T_1_, S’
1421	2.1	–	5.2	0.11	0.17	–	–	–	VIAM	δ’
1423	2.0	–	3.7	0.08	0.15	–	–	–	VIAM	δ’
1430	1.7	1.6	2.7	0.11	–	–	–	–	VIAM	δ’, T_1_, S’
1440	2.4	1.5	0.8	0.1	–	–	–	–	VIAM	δ’, T_1_, S’
1441	1.8	1.8	0.9	0.11	–	–	–	–	VIAM	δ’, T_1_, S’
1450	2.1	2.9	≤0.2	0.12	–	–	–	–	VIAM	δ’, T_1_, θ’
1451	1.7	2.9	≤0.2	0.12	–	–	–	–	VIAM	δ’, T_1_, θ’
1460	2.25	2.9	–	0.11	0.09	–	–	–	VIAM	δ’, T_1_, θ’
Third generation (Li ≤ 2%)									1990s	
2195	1.0	4.0	0.4	0.11	–	–	0.4	–	LM/Reynolds	T_1_, δ’, θ’, Ω
2297	1.4	2.8	0.25	0.11	–	0.3	–	0.5	LM/Reynolds	δ’, T_1_, θ’
2397	1.4	2.8	0.25	0.11	–	0.3	–	0.1	Alcoa	δ’, T_1_, θ’
1461	1.8	2.8	0.5	0.08	0.09	–	–	0.7	VIAM	δ’, T_1_, θ’
									2000s	
2050	1.0	3.6	0.4	0.11	–	0.35	0.4	0.25	Pechiney	T_1_, δ’, θ’, Ω
2055	1.15	3.7	0.4	0.11	–	0.3	0.4	0.5	Alcoa	T_1_, δ’, θ’, Ω
2060	0.75	3.95	0.85	0.11	–	0.3	0.25	0.4	Alcoa	T_1_, θ’, Ω
2065	1.2	4.2	0.5	0.11	–	0.4	0.3	0.2	Constellium	T_1_, δ’, θ’, Ω
2198	1.0	3.2	0.5	0.11	–	0.5	0.4	0.35	Reynolds/McCook	δ’, θ’, T_1_
1469	1.2	3.2	0.3	0.09	0.11	–	0.4		VIAM	T_1_, δ’, θ’, Ω
2076	1.5	2.35	0.5	0.11	–	0.33	0.28	0.3	Constellium	T_1_, δ’, θ’, Ω
2099	1.8	2.7	0.3	0.09	–	0.3	–	0.7	Alcoa	δ’, θ’, T_1_
2196	1.75	2.9	0.5	0.11	–	0.35	0.4	0.35	LM/Reynolds	δ’, θ’, T_1_
2199	1.6	2.6	0.2	0.09	–	0.3	–	0.6	Alcoa	δ’, θ’, T_1_

**Table 6:** Physicomechanical properties of low-weight high-modulus corrosion-resistant weldable Al-Li-based alloys [13].

Alloy Grade	Yield Strength σ_0,2_, MPa	Ultimate Tensile Strength σ_u_, MPa	Relative Elongation δ,%	Density *d*, g/cm^3^	Young Modulus *E*, GPa
1420	≥270	≥420	≥9	2.47	78
1424	≥350	≥460	≥9	2.54	80
1441	≥340	≥450	≥9	2.60	80
1461	≥510	≥560	≥8	2.63	79.5
1469	≥540	≥600	≥8	2.67	79
Al–1.56% Li	46	94	30	–	–
Al–2.45% Li	195	278	6.6	–	–

**Table 7:** The effect of deformation regime on the value of the average size <*d*> of grains that were formed in the course of natural aging—at 20 °C, for 1.5 years—of the deformed alloy 1450.

Regimes of Deformation	Type of the Initial Structure	The Average Size <*d*>, nm	Type of the Final Structure
*n* = 0.25	Fr + Cel	100	NC + SMC
*n* = 0.5	Fr	75	NC + SMC
*n* = 1	Fr	65	NC + SMC
*n* = 5	Fr + NC	60	NC + SMC
*n* = 10	Fr + NC + SMC	75	NC + SMC

Cel—a celled structure; Fr—a fragmented structure.

**Table 8:** The mechanical properties of the 1469 and 1461 alloys after SPD.

Treatment	Hardness			Resolved Modulus *E*, GPa	Plasticity	Stiffness Index, *H*_IT_/*E*
	*H*_M_, GPa	*H*_IT_, GPa	According to Vickers, GPa			
1469, *n* = 1	2.10 ± 0.04	2.79 ± 0.06	2.58 ± 0.05	76 ± 1	0.76 ± 0.01	0.0368 ± 0.0002
1469, *n* = 5	2.38 ± 0.04	3.16 ± 0.05	2.93 ± 0.04	82 ± 3	0.74 ± 0.02	0.0388 ± 0.0006
1469, *n* = 10	2.26 ± 0.04	3.01 ± 0.03	2.78 ± 0.03	81 ± 1	0.76 ± 0.01	0.0355 ± 0.0002
1469, aging at 160 °C, 30 h (FG-structure)	1.57 ± 0.06	1.97 ± 0.07	1.82 ± 0.06	73 ± 1	0.81 ± 0.01	0.0272 ± 0.0008
1461, *n* = 1	1.95 ± 0.02	2.40 ± 0.02	2.27 ± 0.02	90.1 ± 0.9	0.81 ± 0.01	0.0272 ± 0.0001
1461, *n* = 5	2.20 ± 0.07	3.0 ± 0.1	2.8 ± 0.1	68 ± 2	0.71 ± 0.02	0.0443 ± 0.0007
1461, *n* = 10	2.20 ± 0.03	2.87 ± 0.03	2.65 ± 0.03	83 ± 4	0.76 ± 0.02	0.0347 ± 0.0007
1461, aging at 160 °C, 32 h (FG-structure)	1.54 ± 0.03	1.96 ± 0.05	1.81 ± 0.04	75 ± 3	0.82 ± 0.02	0.0262 ± 0.0004

**Table 9:** The mechanical properties of the 1469 and 1461 alloys after SPD and subsequent annealing at 150 °C for 15 h.

Treatment	Hardness			Resolved Modulus *E*, GPa	Plasticity	Stiffness Index, H_IT_/*E*
	*H*_M_, GPa	*H*_IT_, GPa	According to Vickers, GPa			
1469, *n* = 1	2.15 ± 0.01	2.82 ± 0.02	2.61 ± 0.02	85 ± 1	0.77 ± 0.01	0.0333 ± 0.0001
1469, *n* = 5	2.17 ± 0.03	2.88 ± 0.04	2.67 ± 0.03	80 ± 2	0.76 ± 0.02	0.0359 ± 0.0003
1469, *n* = 10	2.19 ± 0.04	2.88 ± 0.06	2.67 ± 0.05	86 ± 2	0.77 ± 0.01	0.0336 ± 0.0002
1469, aging at 160 °C, 30 h (FG-structure)	1.57 ± 0.06	1.97 ± 0.07	1.82 ± 0.06	72.5 ± 0.9	0.81 ± 0.01	0.0272 ± 0.0008
1461, *n* = 1	1.78 ± 0.01	2.26 ± 0.04	2.0 ± 0.03	78 ± 3	0.80 ± 0.02	0.0289 ± 0.0004
1461, *n* = 5	2.10 ± 0.04	2.79 ± 0.08	2.59 ± 0.07	82 ± 3	0.76 ± 0.03	0.0342 ± 0.0005
1461, *n* = 10	2.02 ± 0.03	2.61 ± 0.05	2.41 ± 0.05	82 ± 2	0.78 ± 0.01	0.0319 ± 0.0003
1461, aging at 160 °C, 32 h (FG-structure)	1.54 ± 0.03	1.96 ± 0.05	1.81 ± 0.04	75 ± 3	0.82 ± 0.02	0.0262 ± 0.0004
